# National formative evaluation of the implementation of Martha’s Rule in English acute providers: an ethnographic study protocol

**DOI:** 10.1136/bmjopen-2026-118837

**Published:** 2026-11-11

**Authors:** Lavanya Thana, Beth Fylan, Sally Prus, Jonathan Benn, Charles Vincent, Jennifer Bostock, Luke Budworth, Amy Tallett, Ruth Baxter, Rebecca Lawton

**Affiliations:** 1 Policy Research Unit in Quality, Safety and Outcomes of Health and Social Care (NIHR QSO PRU), Yorkshire Quality and Safety Research Group, Bradford, UK; 2 Bradford Institute for Health Research, Bradford Teaching Hospitals NHS Foundation Trust, Bradford, UK; 3 School of Pharmacy and Medical Sciences, University of Bradford, Bradford, UK; 4 University of Leeds, Leeds, UK; 5 School of Psychology, University of Leeds, Leeds, UK; 6 Experimental Psychology, University of Oxford, Oxford, UK; 7 University of Kent, Canterbury, UK; 8 Research, Picker Institute Europe, Oxford, UK

**Keywords:** Safety, QUALITATIVE RESEARCH, Surveys and Questionnaires, Patient-Centered Care

## Abstract

**Abstract:**

**Introduction:**

In 2024, Martha’s Rule was introduced by NHS England (NHSE) in 143 NHS acute Trusts (phase 1 pilot sites). Martha’s Rule gives inpatients, family and staff the right to an independent clinical review if there is concern about deterioration that has not been satisfactorily addressed by the immediate care team. It also requires staff to obtain information about a patient’s condition from patients and their families at least daily. While Martha’s Rule has potential to make significant improvements in patient care and outcomes, little is known about how best to implement this change. The aim of this evaluation is to examine the micro, meso and macro factors that interact to influence how Martha’s Rule is adapted locally and its impact on patients, staff and other stakeholders.

**Methods and analysis:**

A prospective single embedded case study underpinned by an adapted conceptual model alongside a public awareness survey. Three acute provider sites will participate anonymously in the evaluation. The study will be carried out in two wards and the respective critical care outreach (CCO) team in each of the participating sites. Data collection will involve preliminary informal ‘fact-finding’ interviews with senior staff, general non-participant observations of wards and CCO teams, structured focused observations of relevant clinical events on wards, and staff, patient and family interviews as well as document review. Permission will also be sought from local leads to process site-specific NHSE metrics (to be collected at a national level from all participating pilot sites in the first wave of the rollout of Martha’s Rule). In addition to this fieldwork, a public awareness survey will be conducted in collaboration with Picker and YouGov.

Qualitative data will be analysed iteratively using a framework approach and NVivo V.15. Statistical analysis will be performed on site-specific data from national NHSE metrics and anonymised survey data from respondents in the general population.

**Ethics and dissemination:**

Research ethics committee approval for this study was granted by the Yorkshire & Humber – Leeds West Research Ethics Committee (REC ID 24/YH/0184).

The findings aim to inform ongoing implementation as well as the development of a protocol and logic model for a summative evaluation of sites in the next wave of the rollout of Martha’s Rule. Given the policy relevance and national importance, we plan to disseminate learning from this evaluation widely and as efficiently as possible to stakeholders including policymakers, academics, professionals, patients, public and community groups through a range of mediums and platforms.

**STRENGTHS AND LIMITATIONS OF THIS STUDY**

- This is an ethnographic case study, using primarily qualitative approaches as well as a largely quantitative public awareness survey.
- This independent evaluation is limited to three diverse acute provider sites, however the focus of the evaluation is on depth of understanding rather than exploring variation between sites.
- Patient and public voice throughout the evaluation and all components of the work ensures that their needs and concerns are highlighted in the conduct of and learning from the study.

## Introduction

 Poor clinical monitoring has been identified as a cause of preventable death in 31% of cases.^1^ Despite UK-wide implementation of the National Early Warning Score system that requires the monitoring of vital signs and an escalation protocol should the scores reach a pre-defined threshold, undetected deterioration continues to contribute to patient harm in hospitals. There is both anecdotal and research evidence that patients and families are in a unique position to provide important insight into the detection of deterioration^2 3^ but they do not always speak up and when they do, may be ignored. It was a case of this kind that led to Martha’s rule being developed for implementation in the UK.

The highly publicised findings of the inquest into the death of Martha Mills, aged 13, from sepsis, developed while admitted for an injury to her pancreas, highlighted failures to respond to the concerns of family members, which could have prevented her death. Following this, Martha’s parents, supported by such agencies as Demos^4^ and the office of the Patient Safety Commissioner, called for urgent improvements to patient safety policy across acute provider sites to prevent such avoidable deaths. Martha’s Rule is now a key focus of national patient safety policy designed to ensure that the concerns of patients and their families are taken seriously and actively responded to.

In March 2024, the implementation of Martha’s Rule in 100 NHS Trusts where 24/7 critical care outreach (CCO) was in place, was announced, with implementation commencing from June 2024 in the 143 selected sites. Broadly, Martha’s Rule provides patients and their families, as well as staff, with a widely advertised and easily accessible route to call for a rapid review from an independent clinical review team where they are concerned that a patient is deteriorating and they do not feel that their concerns are being taken seriously by the local team. Martha’s Rule also involves implementing a structured approach to obtaining information relating to a patient’s condition directly from patients and their families at least daily. In the first instance, this will cover all inpatients, except those in maternity, in acute and hospital trusts.

International ‘patient and family activation’ initiatives and policies have previously been implemented, primarily in paediatric services; Ryan’s Rule, Aishwarya’s Rule, REACH, Call for Help and Hear Me in Australia, Condition H(elp) in the United States (US) and Kōrero mai in New Zealand. Such systems provide some evidence supporting the current policy rule. For example, the evaluation of the Condition H(elp) paediatric response service found that all 32 calls over a 2 year period related to a breakdown in communication between families and staff that might have otherwise gone unaddressed.^5^ Gerdik and colleagues^6^ observed that the introduction of a rapid response system for patients and family resulted in a reduction of deaths in one US hospital; findings which were not mirrored when a similar system was implemented for staff.

Studies of these international initiatives also indicate various challenges to adoption, such as in awareness-building,^7 8^ reluctance on behalf of family members to make use of such services due to not wanting to disturb staff,^7 9^ staff concerns about overuse of the service as well as the potential to have a negative impact on family-staff relationships.^7^

Here in the UK, the ‘Call 4 Concern’ (C4C) service was first piloted by the Royal Berkshire NHS Foundation Trust across inpatient services in 2009.^10^ A 7 year long evaluation of this service was conducted by Mandy Odell.^11^ Findings suggested that the majority of calls resulted in ‘appropriate’ activations with twenty per cent of calls resulting in higher levels of care. A small number of calls were also made by staff. More recently, NHS England (NHSE)’s ‘Worry and Concern pilot’ was carried out in seven regional sites in 2023, testing and implementing methods for patients, families and carers to escalate concerns about deterioration using the Call4Concern system and track their views about their well-being into the health record. A report outlining the findings of the realist evaluation of these pilots was pending publication during the drafting of this protocol. Nonetheless, learning from this work informed the development of the current policy rule.

It is based on this that NHSE (2024) set out three specific components of Martha’s Rule to be implemented across the final selected 143 acute provider sites participating in the first wave of the pilot rollout:

1. ‘All staff in NHS trusts must have 24/7 access to a rapid review from a CCO team, who they can contact should they have concerns about a patient.
2. All patients, their families, carers and advocates must also have access to the same 24/7 rapid review from a CCO team, which they can contact via mechanisms advertised around the hospital, and more widely if they are worried about the patient’s condition.
3. The NHS must implement a structured approach to obtain information relating to a patient’s condition directly from patients and their families at least daily. In the first instance, this will cover all inpatients in acute and specialist trusts’.

The operationalisation and implementation of these three components of Martha’s Rule are the focus of this protocol and will be referenced throughout MR1, MR2 and MR3.

### Need for this research and objectives

Despite the strong moral imperative for Martha’s Rule, its implementation is likely to be challenging and may lead to unintended consequences, not least by diverting critical care resources. Moreover, the impact of monitoring a patient’s condition by asking patients or family members themselves has been studied by our own team and found to have potential for the early identification of deterioration, but the evidence base for this is still weak and rapid implementation before it has been adequately tested has the potential to increase inequalities and to become a tick-box exercise that increases safety clutter.^12^

Thus, there are a number of unanswered questions about the potential and actual impact of the implementation of Martha’s Rule for services, healthcare staff and patients/families. The overarching aim of the study is to evaluate the implementation of Martha’s Rule across three acute provider sites over the course of the first phase of implementation. We will examine how micro, meso and macro factors may interact to influence the development of the local innovation(s) and the success of its implementation as well as its impact on various stakeholders within three English NHS Trusts.

The objectives are:

- To employ a conceptual framework to underpin the evaluation and iteratively adapt this over the course of the study based on the emerging findings.
- To determine how the components of Martha’s Rule are operationalised locally and construct delivery models of the routes to identifying deterioration and escalating concerns.
- To use general and focused observations, semi-structured interviews with a diverse range of stakeholders, NHSE metrics and conduct document review to identify what personal, interpersonal, system and wider contextual determinants interact to enable or hinder the implementation of Martha’s Rule as well as its costs and benefits for stakeholders.
- To make recommendations to inform the development of a protocol and logic model for a summative evaluation of sites in the next wave of implementation, as well as to provide learning for policy-makers that will improve approaches to the implementation in the next phase.

To address the aim and objectives of the case study, we developed the following research questions, which will also be addressed:

- Where an intervention that includes components of Martha’s Rule already exists (in the UK and elsewhere) how well is it working? Is there a one size fits all approach or is there a requirement for flexibility? What are the challenges and implications of intervening in this way?
- To what extent has Martha’s Rule been implemented in three pilot sites in England and how has this been done over the course of the first phase of implementation?
- What are the anticipated benefits and costs of implementing Martha’s Rule at the meso and micro level?
- What are the potential challenges and facilitators of delivering the service?
- What are the (potential or) realised unintended consequences?
- Do members of the public, patients and their families know about and understand Martha’s Rule?
- Who activates critical care service outreach teams and how frequently is it used?
- What do patients and family members report to CCO teams?
- How do patients experience escalating for CCO team review and being asked about their condition on a daily basis and does this latter intervention trigger escalation by staff?

The primary domains of focus are: implementation strategies and progress, local operationalisation as well as the barriers and facilitators affecting these processes. The secondary domains are: existing models, public awareness, activation frequency and caller characteristics, costs and benefits as well as unintended consequences. Particular attention will be paid across all domains to equity.

## Methods and analysis

### Study design

A prospective single embedded case study design^13^ is proposed to develop a robust understanding of the determinants of successful implementation of Martha’s Rule as well as the local operational models developed in the first wave of its rollout. Case studies enable the in-depth evaluation of complex innovations whereby multiple factors contribute to behaviour change within a context. The ‘case’ here, delineated as the “*what” or* ‘bounded system’ to be studied,^14^ is conceptualised as the pilot implementation of the policy, Martha’s Rule, in its first year of the rollout. It is situated within the wider societal awareness of and beliefs about this policy as well as the contextual landscape of the national pilot rollout of the policy in the English healthcare system, including a collective subsample of three NHS trusts, and the hospital(s), services and wards within these organisations. The units of analysis are the local operational model(s) of delivery and the micro, meso and macro perspectives of- and factors influencing- the implementation of Martha’s Rule and will provide the lens for comparison and contrast (rather than at site level as in other multi-site case studies).

We will primarily employ qualitative methods to understand the locally developed implementation strategies and operational models as well as to identify the determinants to successful implementation of Martha’s Rule. Qualitative data will be generated from general structured non-participant observations on wards and CCO teams, and focused observations of tasks performed in relation to Martha’s Rule, semi-structured qualitative interviews with key staff, patients and family stakeholders, which are the focus of this protocol. We will also conduct a documentary analysis of relevant local implementation guidance and policies and communication strategies, publicly available information for staff, patients and families, and advertising materials (eg, posters, leaflets, etc), and collate and analyse site specific NHSE metrics to aid the interpretation of and support our qualitative findings.

A survey will be developed and undertaken within the life of the evaluation in collaboration with Picker to elucidate macro (unit of analysis) perspectives and factors contributing peripherally to the implementation of Martha’s Rule within the immediate NHS settings. The aim of the survey will be to understand public awareness of Martha’s Rule in each of the local regions of the three English NHS sites. The survey will be run by Picker and YouGov using a Care Opinion sample and YouGov panel in line with the rollout of Martha’s Rule and national comms about the policy. The survey results will be synthesised alongside other findings of the overarching evaluation.

The overarching study will commence in August 2024 following the official implementation of Martha’s Rule in June 2024 and run until June 2026. An updated systematic review (PROSPERO ref: CRD420251013254), informal preliminary groundwork (eg, fact-finding interviews) and document review will be conducted in the early phase of the study. The case study evaluation, involving iterative formal qualitative and quantitative data collection and data analysis, will run from November 2024 to June 2026. The end of the evaluation will coincide with the final member-reflection session with members of the Public Advisory Group and completion of data analysis in June 2026.

### Framework approach

A theoretically driven deductive qualitative methodology, a framework approach, was chosen to underpin the study design from its outset.^15^ Figure 1 provides an overview of the adapted conceptual model underpinning the study, which includes the locally developed model for operationalising Martha’s Rule and associated tasks performed by stakeholders, the primary contexts for implementation, the implementation process, and the determinants to implementation that we are seeking to describe and/or measure. The framework has been adapted from the Consolidated Framework for Implementation Research (CFIR)^16^ and the Systems Engineering Initiative for Patient Safety V.3.0.^17^ Given the anticipated potential for components of Martha’s Rule to widen inequities in healthcare, we also incorporated the three health equity domains from the Health Equity Implementation Framework:^18 19^ (1) culturally relevant factors such as stakeholder characteristics and implicit bias, (2) the clinical interaction between healthcare professionals and patients and potential for bias and discrimination and (3) the wider societal context including physical environments (eg, access to translated patient information) and sociopolitical forces such that influence structural factors that perpetuate oppression.

**Figure 1:**
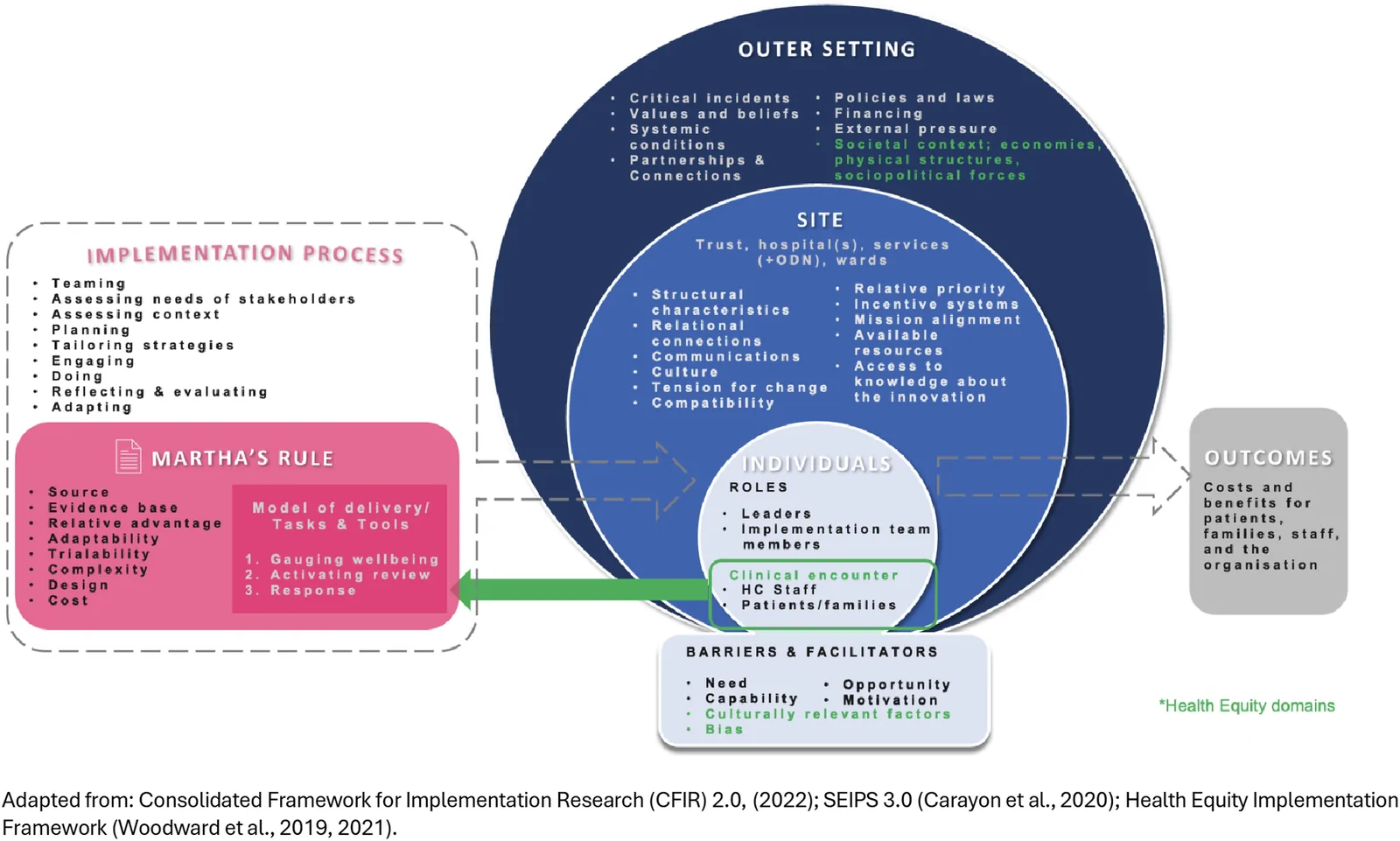
Adapted conceptual framework. This conceptual framework underpinning the evaluation was primarily adapted from the Consolidated Framework for Implementation Research (CFIR) 2.0 (2022),^25^ and applies elements of Systems Engineering Initiative Patient Safety (SEIPS) 3.0^17^ and the Health Equity Implementation Framework.^18 19^ It incorporates five primary domains: the outer setting, the site, individuals, the implementation process, and outcomes. Onto these layers are mapped the intervention, Martha’s Rule, the models of delivery, tasks and tools,^17^ as well as health equity domains such as culturally relevant factors.^18 19^

We will use the conceptual framework to flexibly and deductively guide our data collection and analysis while allowing for inductive insights to be generated and shape the framework as iterative analysis progresses. Our primary focus within the framework will be to elucidate the antecedents to implementation, the locally developed/adopted delivery model, the implementation process, roles of the individuals and the clinical encounter, as well as the impact on stakeholders’ roles (costs and benefits) and the barriers and facilitators to implementation within the ward and hospital level contexts. Although we will pay attention to factors in the outer setting and system level settings (eg, hospitals) where these influence implementation, it may not be possible within the scope of this formative evaluation to fully explore all of the constructs detailed within these layers.

### Case study setting and sites

Fieldwork, including non-participant observations and semi-structured interviews involving staff and patients and families, will primarily take place on the premises of the three acute providers participating in the evaluation, with some possibility for remote interviews where necessary.

### Site selection and anonymity

Three English NHS acute provider sites have expressed interest in participating in the formative evaluation. Two of these will be large urban teaching hospitals with a diverse local population and the other, a district general hospital. All NHSE pilot sites were required to have an established 24/7 CCO team covering all adult inpatient wards. To ensure some aspects unique to paediatric critical care are incorporated in the study findings, we will include at least one site that will have remote paediatric critical care outreach (PCCO) coverage provided by an Operational Delivery Network (ODN) – the process of selecting these ODNs and the sites they serve is currently underway by the Health Innovation Network and will be confirmed imminently. We will maintain the anonymity of the three sites participating in the evaluation through the course of the study to ensure the study’s integrity, stakeholder confidentiality, and to minimise potential bias. Therefore, only members of the core research team will be aware of the selected sites.

We plan to carry out the study in two wards in each of the three participating sites. We will also observe the CCO team in each site, including remote coverage of paediatric critical care provided by the ODN for at least one site.

A detailed overview of the procedures at sites and consenting processes can be found in online supplemental material 1) along with figure 2 illustrating formal data collection.

**Figure 2:**
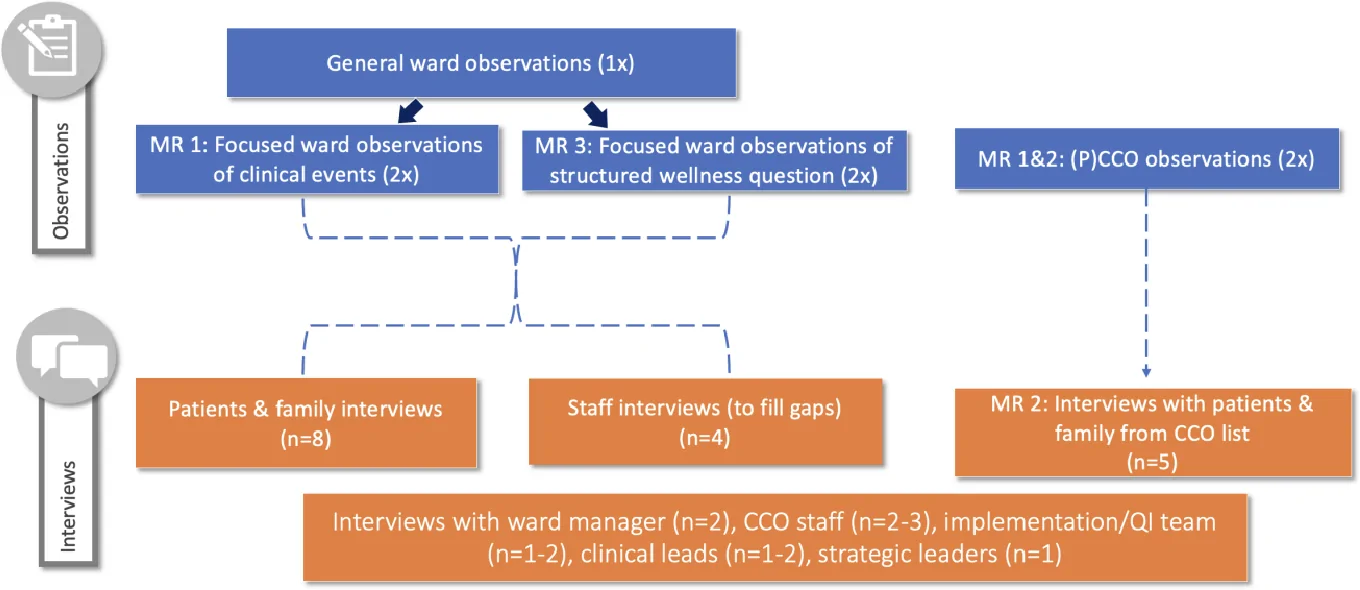
Formal observations and interviews at each site. This figure illustrates the flow of research observations and interviews to be conducted at each site. It also provides the number of times a set of observations will be conducted over the course of the evaluation (eg, twice) and the number of each type of interview with different stakeholders per site.

## Data collection from sites

### Observations

#### Preliminary meetings

Prior to commencing observations, researchers will meet with the CCO manager and ward managers of the selected wards at each site to introduce the study, seek their permission for the observations to be carried out, gain a preliminary understanding of the context (eg, number of beds and bays, types of patients and conditions, constitution of the multidisciplinary clinical and care team), and build rapport. During these meetings researchers will establish clinically relevant routines on each ward and the CCO team in relation to the operationalisation of Martha’s Rule and agree on clinical events and timepoints for focused observations.

#### General observations

Non-participant observations of the identified wards will be carried out prior to commencing focused observations. Researchers will engage in informal conversations with various staff on wards, observe the general physical environment and clinical routines to gain an initial understanding of the context, culture, usual patterns of working and key interactions between staff that will be of relevance to operationalising Martha’s Rule. Anonymised notes will be taken on the physical context, the processes involved in carrying out routine clinical practices and patterns of working, as well as the relevant interactions between staff at all levels (from consultants to healthcare assistants HCAs) in carrying out clinical care. These observations will be carried out over 1 week (4–5 days, approximately 20 hours) on each ward at one time point at the start of the formal data collection phase.

*MR component 1&2:* Due to the anticipated relatively low level and spread of calls to CCO teams (based on previous studies^6 10 11^) it may not be logistically possible to observe these in practice. However, we will schedule observations or the ‘shadowing’ of CCO staff over the duration of their shifts to observe the general culture of team working in the CCOs and, where possible, be present at times when call rates are higher to observe the process of receiving, triaging and responding to patients of concern on wards whether or not these are activated through Martha’s Rule. These observations will occur two times over the course of the implementation process.

#### Focused observations

Following general observations, focused and structured non-participant observations on wards will be conducted by researchers.

*MR component 1&2:* brief observations will be conducted at key clinical events relevant to the operationalisation of Martha’s Rule which were previously identified with ward managers. This may include routine ward rounds (eg, early morning, mid-morning and afternoon), during medicine rounds, and/or handovers (at end of shifts) and/or mid-shift ‘safety huddles’ where potential and actual deteriorating patients are likely to be flagged and escalated. Relatives may also call in to the ward, be invited to attend, or be updated around the time of these rounds. Each observation will be conducted for the duration of the event, which could last up to several hours. To allow for comparison, observation times (eg, time of day, weekend) will be varied. These observations will take place at two timepoints over the course of implementation of Martha’s Rule progresses.

*MR component 3:* after establishing the ward adopted method for asking patients about their condition or well-being (eg, using the Patient well-being Question(naire)^3^ on at least a daily basis) researchers will undertake focused non-participant observations at these key timepoints (eg, during ‘intentional rounding’ or routine tasks performed by HCAs). Researchers will also observe nursing staff and HCA interactions with patients at these events, including particularly where a patient there has been concern about a patient deteriorating. We will take fieldnotes on how responses to well-being questions are recorded, and how changes in well-being are identified and tracked (eg, via Electronic Patient Record, scoring tool, etc), and where possible, how and when these well-being indicators of deterioration are then escalated to other ward staff and/or the CCO team.

Informal conversation will be had with staff during observations, for example to understand the culture on the ward and rationale for carrying out certain tasks and procedures in relation to Martha’s Rule.

Structured templates for recording fieldnotes will be developed by the research team following fact-finding interviews and preliminary meetings with ward managers and in conjunction with review of the conceptual framework for the evaluation to ensure consistency of notes across sites, wards and researchers. Each observation of individual clinical interactions will be brief and last up to a maximum of 1 hour. The researcher will observe several clinical interactions during an observation period and have informal conversations with staff. These will occur over two timepoints as implementation of Martha’s Rule progresses.

Observation tools used to support data collection during observations are provided in online supplemental material 2). These tools were developed and refined in the field and therefore were not submitted as part of the original ethics application.

#### Semi-structured interviews

This qualitative study will involve the use of semi-structured interviews to enable participants to share their experience of operationalising components of Martha’s Rule. The aim of the patient and family interviews is to gain understanding of individuals’ experiences of and the responses to deterioration and the barriers and facilitators and acceptability of the avenues to escalate their concerns and receive an independent review outside of their base teams. The aim of individual staff interviews is to further understand how the components of Martha’s Rule have been operationalised and implemented locally, the barriers and facilitators to implementation, as well as their acceptability.

Most patient and family interviews are likely to be completed on wards where patients are well enough to participate and where it is possible to ensure privacy and confidentiality. We will also offer interviews via telephone or video conference (Zoom, Teams, Skype or Facetime) or telephone once the patient is discharged home. Staff participants will be offered a face-to-face on-site or a remote interview at a convenient time for them.

Patient and family participants may request to have an ‘assisted interview’ whereby the participant chooses to have a friend or relative present for emotional support or has language or other communication barriers (eg, due to hearing impairment) and requires some assistance to complete the interview. A spoken language or British Sign Language (BSL) interpreter can be provided by the research team if needed for remote interviews. A second follow-up interview may be arranged with the participant’s consent if it is necessary to gather further information or, for safeguarding reasons, to split the interview into two sessions.

All interviews should last between 30 to 60 min and will consist of mainly open-ended questions. In exceptional cases, this may be extended to enable participants to share their experience at their own pace and to best suit the individual participant’s needs (eg, to allow time for any breaks required due to disability). Interviews will be audio-recorded using built-in video-conferencing software (Zoom) or audio-recorded via an encrypted dictaphone. Audio-recordings will be sent for verbatim transcription by a professional transcriber. Transcripts will be checked and de-identified.

All staff, patient and family member participants will be offered a £15 shopping voucher and staff to thank them for their time.

#### Interview schedule development

Using a semi-structured approach to interviewing, the researcher will use the interview schedules flexibly to ask questions as well as using open prompts to enable the participant to share their experiences and perspectives in a way that is meaningful to the participant while addressing the research questions of the evaluation. Interviews will focus on specific details associated with the experience of the individual’s role in relation to operationalising and/or implementing Martha’s Rule, perceptions of the service, organisation and wider societal context where these have impacted the participant’s experience or perception of Martha’s Rule, and how factors across the healthcare system may interact to influence behaviours.

The interview schedules have been developed by the research team based on the adapted conceptual framework for the evaluation and refined with input from patient and public involvement (PPI) members. Prompts will be used to elicit inductive data to be generated. The schedules will be piloted with a small number of stakeholder participants at the outset of the study and further refined as needed. Interview schedules are provided in online supplemental material 3).

## Interview sample and recruitment

### Interviews

At each site, we will conduct approximately one preliminary informal interview with system leaders, one with implementation team members, one with operational leads and one with clinical leads for Martha’s Rule in the early phase of the work, resulting in around four interviews at each site and 12 across the three sites during informal data collection.

On formal data collection, semi-structured interviews will be undertaken with a purposively selected sub-sample of approximately 3–4 patients and family members on each ward. Assuming two wards per site will be participating in the evaluation, this would give approximately eight interviews per site, and 24 across all three. We will interview a further 5 patients and family members per site from CCO lists of those activating a rapid response, resulting in 15 across all three sites. We will have informal conversations with staff during and following observations. Where it is necessary to fill in any gaps in understanding and provide privacy, we will conduct a small number (approximately 3–4) of follow-up interviews with staff members (clinical and support front line staff) participating in observations on each ward; giving 12 additional interviews. We will also interview ward managers on each ward (2), CCO team members (2–3), implementation development/quality improvement team members (1–2), clinical and operational leads (1–2), strategic leaders (1) per site. Resulting in 10 further interviews in each site and 30 across the three. In total, this would mean approximately 80 further interviews will be conducted in total across the three sites during formal data collection.

These numbers are likely to be needed to ensure that each perspective, at the micro, meso and macro (where possible) level, can be adequately understood in terms of the meanings attributed by stakeholders, the receipt of care by various patients and families, as well as the process of the operationalisation/delivery of components of Martha’s Rule. Ultimately, this relies on judgement since there are no hard and fast rules determining sample size estimates for qualitative research. As the field work and analysis progresses, it may become possible to assess whether the prior estimates of the numbers of interviewees needed in each group can be amended based on achieving a sufficient level of confidence in data ‘saturation’.

Purposive sampling will be used to recruit a diverse sample of patient and family interviewees at site level, including: experience of being asked about their condition on a regular basis; whether or not they activated an independent rapid review via the local escalation service (eg, Call4Concern); and a range of demographics (eg, gender, ethnicity, level of education, disability, etc). We will ensure that minoritised people and lower sociodemographic groups are represented adequately in the study.

Researchers will make contact with local (to the specific site) community group organisers (such as ethnic, religious groups or disability and learning difficulties charities) or key members of the organisation to ensure that a range of considerations and needs are accommodated for in recruiting a diverse sample from the participating wards. Initial contact will be made with these groups via telephone or in person, where possible, using previously established links within the research team and our Public Advisory Group. Suitably adapted recruitment materials, developed with input from the PPI members, will be used and adapted recruitment advertisements (eg, language translated versions) will also be posted on the selected wards at each site.

### Selection criteria

#### Staff participants

We will speak to frontline staff informally during observations and may invite a small number carrying out roles in relation to the implementation of Martha’s Rule (eg, asking the structured question about patient’s condition) at each site to participate in an interview. This could involve: HCAs, nurses, junior doctors, nurse in charge, ward managers, consultants and CCO team members. We will also interview other staff involved in the implementation of Martha’s Rule as follows: implementation team members, operational and clinical leads, and strategic leaders.

Inclusion Criteria

- Employed by or a casual worker (eg, bank staff) at the participating English NHS Trust.
- Aged 18 or over.

#### Patients and family participants

We will use purposive criterion sampling to identify the patient and family participants from the sites on the following primary criteria:

#### Inclusion criteria

- Person who has either: (1) Had first-hand experience of being asked about their condition on a ward, (2) Had first-hand experience of accessing an independent rapid review of their care during an inpatient stay by the PCCO team OR (3) Is a family member of an adult or child who has experience of 1. Or has first-hand experience of accessing a rapid review of their loved one’s care by the CCO team.
- Aged 18 or over.
- Has capacity to provide informed consent.

#### Exclusion criteria

- The person who experienced inpatient care is deceased.
- Insufficient comprehension or attention to be able to engage in an interview.

In instances where a participant is not proficient in spoken English or is hearing impaired, they may request for either a friend or relative to support them in communicating their experience in an assisted interview, or an NHS approved language or BSL interpreter to be provided by the research team. These are distinct from family member interviews, during which a carer will be interviewed alone about their own experience of escalating their concerns and activating a rapid review for a patient who has deteriorated on a ward.

#### Purposive sampling

We will seek to obtain a maximum variation of the sample. A sample size of up to 80 participants across the three sites was based on the number necessary to reflect a range of stakeholder experiences of components of Martha’s Rule across various healthcare settings as well as staff and patient and family participants’ diversity (in age, gender, ethnicity, socioeconomic status etc). In accordance with the study methodology, we anticipate interviewing up to 80 participants, or until a sufficient degree of data saturation has been achieved to elucidate key components within our conceptual framework.^20^ A record of efforts to diversify the sample will be maintained and tracked as data collection progresses to ensure diversity and enhance the transferability of the study’s findings.^14^ We will remain flexible about the proportions recruited across participant groups and sites to sufficiently meet requirements of the framework analysis, but will endeavour to include a large enough number of people from minoritised groups (20%–25% of the sample at each site).

#### ‘Screening’

Short questionnaires will be completed with potential interview participants to ensure there are sufficient numbers of participants from each of the settings and across different demographic groups as data collection progresses, and to enable consistent reporting across the entire sample. Using the screening questionnaire we will collect basic details of the ward (eg, respiratory or ward number), demographic information, and experience of components of being asked about their well-being as well as decision-making undertaken in contacting the local escalation service. Participants who do not meet eligibility criteria will be immediately informed that they do not meet criteria for participation in the study, although we do not anticipate this occurring often due to the procedures for recruitment (eg, identified in collaboration with staff from wards).

## Site-specific NHSE metrics

Permission from local leads at the three sites will be sought to process and analyse anonymised site-specific data collected by NHSE as part of their monitoring audit of the first wave of implementation of Martha’s Rule across all 143 participating pilot sites. These metrics will be used to assist with the interpretation of and support our qualitative findings. The process of determining and defining these metrics at a national level is currently underway. Data used in the evaluation will likely include: number of calls to the escalation response service from patients, families and staff; number of inappropriate calls; gender, age, ethnicity, socioeconomic status of patient as well as any disability/impairment; language used by caller to escalate; percentage of wards for which each component of Martha’s Rule has been implemented; number of occasions that a patient wellness score triggers a change in treatment pathway; number of calls to escalation response service triggers a change in treatment pathway.

## Public survey

In partnership with our National Institute for Health and Social Care Quality Safety and Outcomes Policy Research Unit collaborators Picker (https://picker.org), we will run a public survey of awareness of Martha’s Rule and views of safety in hospitals and speaking up about concerns more broadly. The survey will allow us to elucidate wider society perspectives and factors (macro unit of analysis) contributing peripherally to the implementation of Martha’s Rule within NHS settings. The aim of the survey will be to understand public awareness of Martha’s Rule and views on patient safety in each of the local regions of the three English NHS sites. The survey will be administered online by (1) a sub-contractor, YouGov (https://yougov.co.uk), a global leader in public opinion surveys and (2) Picker, an independent health and social care research charity specialising in national healthcare surveys.

### Setting

The YouGov survey will involve their panel members from the general population within two local regions in which our three anonymous NHS sites are located.

The Care Opinion survey, administered by Picker, will be recruited from those who have given consent to be contacted for research purposes following the submission of their patient ‘stories’ and feedback of English healthcare on the Care Opinion platform.

### Survey development

A survey has been developed by the research team based on relevant literature and in collaboration with our Patient Advisory Group comprising lay members of the public with relevant lived experience. It subsequently underwent two rounds of cognitive testing by our partners at Picker with a diverse (eg, ethnicity, gender, region, disability, etc) sample of 10 participants recruited from the general population, including some people with either prior inpatient experience or prior experience of supporting a friend or family member in hospital. The questions were further refined based on their feedback. The survey is provided in online supplemental material 4).

This is a 23-item survey that progressively elicits responses on general experience of hospitals and feeling of safety to more specific questions relevant to MR3, the structured wellness question and MR2, access to and experience of a service to escalate concerns. The inclusion of ‘filter questions’ means that 12 items are answered by all respondents. The survey questions ask about any previous experience of hospital care as a patient or friend or family member supporting a patient, views of general safety in hospital, any concerns that patients and their family or close friends might experience during an overnight stay in hospital, speaking up about any such concerns, particularly about themselves or loved ones getting worse in hospital, as well as peoples’ awareness of and perspectives on Martha’s Rule. This version of the survey will be completed by both YouGov panel respondents, and four additional questions that expand on former questions will be answered by the Care Opinion sample of people in the community.

Alongside our survey questions, YouGov use standard demographics questions (eg, age, gender, region, disability) that will also be asked of the Care Opinion sample. We have also developed additional demographic questions on ethnicity and main source of national news that have been included for both samples. These questions also underwent cognitive testing and were refined. Demographic data will be included in the full dataset for analysis.

The survey wording will be further scrutinised by YouGov and Picker to ensure it meets their quality standards, before running the surveys with the YouGov panel and Care Opinion sample respectively. This may result in minor adjustments to the questions or response options with the agreement of the research team.

### Survey samples and recruitment

The survey will be run by the sub-contractor, YouGov, and by our partners at Picker using the YouGov panel members and a Care Opinion sample respectively. *We will not recruit any survey respondents from NHS sites or services directly*. However, to enable us to contextualise our qualitative findings, the YouGov sample will involve respondents in the geographical regions in which our three NHS sites are located.

### Survey sample recruitment

Our survey respondents will comprise two samples; one involving a YouGov panel and the other from Care Opinion.

#### YouGov panel

The sub-contractor, YouGov, has carefully recruited a panel of millions of people to take part in their surveys. Panel members are recruited from a host of different sources, including via standard advertising and strategic partnerships with a broad range of websites. YouGov will conduct the public opinion survey online via “active sampling”. The emphasis is always on the quality and representativeness of the sample rather than the quantity of respondents.

The survey sample size of the YouGov sample is estimated to be 2000, spread equally across two regions in which our three anonymous acute NHS sites are located. A representative sample of their panel members in the general population will be recruited by YouGov.

#### Care opinion sample

Respondents will also be recruited from a public database of patient stories held by Care Opinion (https://www.careopinion.org.uk). Care Opinion is a leading national non-profit online patient feedback platform where patients are able to share their health and social care experiences and provide feedback.

A purposive sample will be recruited using those who have shared their experiences with Care Opinion within the last 3 years and given consent to be contacted by Care Opinion for other research. A list of those in the Care Opinion database will be generated by Care Opinion based on additionally filtering for those with experience in acute specialities (eg, general medicine, general surgery, urology, gynaecology, cardiology etc) in England. Care Opinion will contact people on this list directly with an email invitation containing a link to take part in the online survey. No identifiable data will be shared with Picker or the research team.

The survey sample size of 300 is estimated based on Care Opinion’s advice about response rate and inviting people from this list of approximately 3000 potential respondents (based on running preliminary searches using filters on the Care Opinion database) to take part in the Picker survey. If a lower than anticipated number of respondents complete the survey, the filters will be expanded and a further invitation will be sent out to those in this wider list.

#### Inclusion criteria

The inclusion criteria are the same for both survey samples. Respondents can take part in the survey regardless of prior experience of inpatient care or supporting a loved one in hospital.

- Aged 18 or over.
- Living in England.

#### Exclusion criteria

The exclusion criteria are the same for both survey samples.

- Aged below 18 years.
- Living outside of England.

### Data collection

The survey will be administered online by YouGov to their panel members and by Picker for the Care Opinion sample. Data will be collected anonymously. The survey will be hosted by YouGov using their online survey platform and panel members and by Picker in Qualtrics using a Care Opinion sample. The data collected will be in anonymised format. The survey items will be included verbatim in both surveys; however, the Care Opinion sample will ask an additional four questions. The full survey will take approximately 15 min to complete.

Anonymous survey responses will be collated and cleaned by the sub-contractor, YouGov, and Picker for each respective sample. YouGov will provide Picker and the research team with the raw dataset and analysed tables for the YouGov sample. Picker will provide the research team with the raw dataset for the Care Opinion sample.

## Data analysis and member-checking

Participant interview data, structured fieldnotes of observations and preliminary ‘fact-finding interviews’, as well as relevant documents, will be analysed using a framework method^21 22^ to elucidate the implementation strategies, intervention components (and build operational models) and determinants of successful implementation. This structured matrices approach to analysis will allow for the units of analysis (micro, meso and macro factors and delivery models) in this case study to be transparently elucidated and exhibited, and is amenable to both deductively and inductively generated themes.^21^ The study’s conceptual model and, in particular, the constructs of the CFIR,^16^ will primarily dictate the higher order domains (intervention components, implementation process, individual characteristics, inner setting and outer setting) for the matrix.

Gale and colleagues^21^ detail seven stages in the process of framework analysis. The first two steps involve transcription of audio recordings and familiarisation with the data sources, of which there are a variety in this study: interview transcripts, observation fieldnotes and relevant documents and materials. The field researchers will work with the lay analyst and wider team to undertake the analysis. The process will commence with conducting an initial line-by-line coding of each data source whereby each analyst provides descriptive labels which define phenomena using an inductive process of coding. Alongside this, team discussion and development of a framework or coding tree for analysis will be undertaken. The framework will support deductive coding using pre-defined codes and criteria, using constructs from the study’s conceptual framework and the CFIR,^16^ as well as any prominent inductive codes. Once agreed, the coding framework will be used to index other data sources, and a spreadsheet will be used to chart or summarise data into an overarching matrix. Connections and comparisons of categories and between micro, meso and macro perspectives will be made in interpreting the matrices. Framework analysis is compatible with constant comparative methods^23^ - simultaneous, iterative and cyclical data gathering and analytic processes – enable emergent categories or social patterns and mechanisms^24^ to be systematically ‘abstracted’.^23^ Throughout the analytic process the researchers will write reflective ‘memos’ or descriptions of the codes and categories and their relationships to one another. These memos enable the researchers to think creatively, generate new ideas about the data and identify areas for further data collection.

The steps involved in the analytic process are iterative alongside data collection, whereby the researcher engages with the data, describes, analyses and charts the data, identifies gaps in the chart then returns to the field to collect further data to be analysed. We plan to train and support a lay/PPI analyst to contribute to the team analysis from the initial coding stage. Team analysis meetings will be set up and run periodically throughout the data collection and analysis period to ensure agreement of the coding frame and definitions and to reflect on the developing matrices and interpretation of the data.

### Quantitative data analysis

#### NHSE metrics

This strand characterises Martha’s rule implementation and call activity and supports the qualitative findings. NHSE monitoring data are aggregate counts at site and month level, returned over the course of the evaluation. The data metrics collected, by site and month:

- Implementation coverage (wards implemented as a percentage of wards in scope, by ward type and component, including the patient wellness questionnaire).
- Call volume by caller type (patient, family or carer, staff).
- Qualifying status, with recorded reasons for non-qualifying calls.
- For qualifying calls:
  - The outcome (advice, intervention, transfer, other).
  - Demographics: patient ages (number of calls per age bracket), gender (n calls per gender), plus number of calls per ethnicity categories and deprivation decile.
  - Early warning score before the call and at review.

Metrics will be summarised as counts, proportions and rates with confidence intervals (CIs), and described and plotted over time. Where enough data have accrued, we will test change over time and compare activity before and after implementation at each site, using count models that allow for differences between sites.

Early review of data has found demographic related variables including deprivation, ethnicity, language and early warning score fields (recording only for qualifying calls) have a lot of missing data. Formal statistical testing will only be carried out where the numbers are large enough to support it; where they are not, including for the patient level breakdowns of qualifying calls (eg, by demographics), results will be described rather than tested. Planned breakdowns are by site, caller type, qualifying status and the patient characteristics above; with only a few sites involved, differences between sites will be described rather than tested.

We will distinguish months with genuinely no calls from months where a site simply did not report, note how complete each site’s data are, and repeat the main analyses using only the fully reported months.

#### Survey

Public awareness will be estimated from two separate surveys: an online panel survey (YouGov) covering the two regions, and a survey run by Picker with people recruited through Care Opinion, covering England.

The two were designed somewhat differently and are not directly comparable, so they will not be combined. We will analyse the raw responses from both and adjust each to match the make-up of the population (eg, by age, gender, region, income and education) reporting both the unadjusted and adjusted figures.

For a prespecified set of key survey items addressing the research questions on public awareness, comprehension and acceptability of Martha’s Rule, we will report the percentage answering each way in both surveys, with CIs, and the size of any difference between them. These items will be selected before formal analysis begins and reported in the supplementary materials. We will also report these by key sociodemographic groups (eg, age, gender, ethnicity, region, income and education).

Because Care Opinion covers all of England and YouGov only two regions, an England-wide estimate will come from Care Opinion, with YouGov used as a regional comparison.

Missing data will be handled by complete case analysis, with multiple imputation as a robustness check.

### Patient and public involvement (PPI) and member reflections

We will work closely with our Public Advisory Group comprising lay members with relevant lived experience throughout the evaluation. This group previously inputted into the design of the study and development of the participant-facing materials. We plan to involve and support lay peer researchers to contribute to team analysis of anonymised qualitative data to aid insights, especially in relation to the patient and family interviews and for particular sections of the population.

‘Member-reflection’ sessions will involve the Public Advisory Group at interim stages in the iterative data collection and analysis phases to ensure that the findings generated by the researchers have real-world resonance for PPI members who have lived experience of inpatient care and deterioration and escalation of their concerns. Input and feedback from three planned member-checking sessions over the course of the study will be considered and incorporated in the subsequent return to data collection and analysis, and in the write-up of study findings.

## Ethics and dissemination

###  Ethical approval

Research ethics committee approval for this study was granted by the Yorkshire & Humber – Leeds West Research Ethics Committee (REC ID 24/YH/0184). A substantial amendment for the survey component of the research was submitted in June 2025 and approved by the Yorkshire & Humber – Leeds West Research Ethics Committee on the 25 June 2025.

This study is focused on a sensitive area involving patient deterioration and it will be important to ensure its conduct protects both participants and researchers from any negative consequences. All co-applicants have extensive experience of conducting research on potentially sensitive health-related topics. However, we will particularly use the expertise of our lay colleagues as well as research team of patient safety experts to plan the fieldwork in such a way as to minimise anxiety and enable trust.

Sample recruitment materials and the survey were developed with feedback from lay members, to ensure these are sensitive and provide reassurance on confidentiality, offer a choice of location for interview, ability to opt out of observation, the possibility of being interviewed together with a family member or other advocate and information on sources of support that can be accessed if the interview or survey leads to emotional distress. We will have in place safety netting processes to ensure any participants or research staff can seek extra support if needed to deal with emotions that may be activated by the research.

### Dissemination

Throughout the evaluation, we will ensure that our close and collaborative relationship with NHSE Deterioration Programme team is maintained, which is a strength and fundamental to ensuring that this research drives policy change. Learning will also be used to inform the further development of a logic model of Martha’s Rule and a protocol for a large-scale summative evaluation. We will produce academic publications and conference abstracts, plan other dissemination activities, including video summaries of learning for various audiences. To disseminate learning from the evaluation as widely as possible we plan to work with our partners to include the NHSE, National Institute of Health and Social Care Research, Department of Health and Social Care, National Directors of Patient Safety, the Patient Safety Commissioner, our Public Advisory Group, Patient safety partners and various patient and community charities and organisations.

## Supplementary material

10.1136/bmjopen-2026-118837 online supplemental file 1

10.1136/bmjopen-2026-118837 online supplemental file 2

10.1136/bmjopen-2026-118837 online supplemental file 3

10.1136/bmjopen-2026-118837 online supplemental file 4

## Data Availability

No data are available.
