# Supplementary material for: National formative evaluation of the implementation of Martha’s Rule in English acute providers: an ethnographic study protocol
Source: BMJ Open. 2026 Nov 11;16(11):e118837. doi: 10.1136/bmjopen-2026-118837 (PMC13756334; doi:10.1136/bmjopen-2026-118837)
Supplement: Supplementary file 1 [file bmjopen-2026-118837.reviewer_comments.pdf]

PEER REVIEW HISTORY

BMJ Open publishes all reviews undertaken for accepted manuscripts. Reviewers are asked to complete a checklist review form and are provided with free text boxes to elaborate on their assessment. These free text comments are reproduced below.

ARTICLE DETAILS

Title (Provisional)

A national formative evaluation of the implementation of Martha's Rule in English acute providers: An ethnographic study protocol

Authors

Thana, Lavanya; Fylan, Beth; Prus, Sally; Benn, Jonathan; Vincent, Charles; Bostock, Jennifer; Budworth, Luke; Tallett, Amy; Baxter, Ruth; Lawton, Rebecca /

VERSION 1 - REVIEW

**Reviewer**	**1**
**Name**	**Koike, Daisuke**
**Affiliation**	**Nagoya University Hospital, Department of patient safety**
**Date**	**30-Apr-2026**
**COI**	**None**

Thank you for the opportunity to review this study protocol. This is a timely and important evaluation of the implementation of Martha's Rule, addressing a policy-relevant patient safety initiative. The protocol is generally well structured and provides a detailed overview of the planned formative evaluation, including qualitative fieldwork, survey components, and analysis of implementation-related metrics. However, several aspects of the methods and evaluation outcomes would benefit from further clarification to improve transparency, reproducibility, and interpretability.

Major comments

Interview guide

The overall methods are described in sufficient detail; however, to improve transparency and reproducibility, the authors should provide the interview guide, or at least examples of the main questions and prompts, as supplementary material.

Questionaries

The survey component is described in terms of development process, sample, and administration; however, the actual survey items and response options do not appear to be

provided. Including the survey instrument as supplementary material would improve transparency and reproducibility.

Field note

The procedures for observations and fieldnote-taking are generally described, including the use of anonymised notes and the planned development of structured templates. However, the actual fieldnote template or examples of key observation domains are not provided. Including the fieldnote template, or at least the main domains to be recorded during observations, would improve transparency and reproducibility of the qualitative component.

Qualitative analysis

The authors state that the qualitative data collection and analysis will be guided by an adapted conceptual framework based on CFIR, SEIPS 3.0, and the Health Equity Implementation Framework. However, it is not fully clear how these frameworks will be operationalised into specific observation domains, interview questions, fieldnote items, or initial coding categories. Providing further detail, such as examples of observation domains, or an initial coding framework, would improve transparency and reproducibility.

Outcome

The protocol includes a wide range of evaluation targets, including local operationalisation, barriers and facilitators, costs and benefits, unintended consequences, public awareness, activation frequency, caller characteristics, and treatment pathway changes. While this breadth is understandable for a formative implementation evaluation, the outcomes or evaluation domains appear somewhat overextended and are not clearly prioritised. Clarifying the main evaluation domains and distinguishing core outcomes from exploratory or contextual measures would improve clarity and interpretability.

Statistical analysis

Several aspects of the quantitative analysis plan remain somewhat vague, including the final NHSE metrics to be used, the definitions of key variables, the criteria for conducting inferential analyses, and the handling of subgroup analyses and missing data. These points should be clarified to improve transparency.

VERSION 1 - AUTHOR RESPONSE

Reviewer 1 comments:

Interview guide<br>The overall methods are described in sufficient detail; however, to improve transparency and reproducibility, the authors should provide the interview guide, or at least examples of the main questions and prompts, as supplementary material.	There are a number of interview guides (five) used for interviews with various stakeholders. We have collated these for inclusion as supplemental material file 2., provided with this response.

Questionaries<br>The survey component is described in terms of development process, sample, and administration; however, the actual survey items and response options do not appear to be provided. Including the survey instrument as supplementary material would improve transparency and reproducibility.	The survey items developed by the study team have been included in full for supplemental material 3.
Field note<br>The procedures for observations and fieldnote-taking are generally described, including the use of anonymised notes and the planned development of structured templates. However, the actual fieldnote template or examples of key observation domains are not provided. Including the fieldnote template, or at least the main domains to be recorded during observations, would improve transparency and reproducibility of the qualitative component.	There are multiple observation tools used for different general and focused observations. These were not submitted for ethical approval - the study team developed and refined these in the field as the data collection progressed. We have provided the 5 observation tools as supplemental material 1.
Qualitative analysis<br>The authors state that the qualitative data collection and analysis will be guided by an adapted conceptual framework based on CFIR, SEIPS 3.0, and the Health Equity Implementation Framework. However, it is not fully clear how these frameworks will be operationalised into specific observation domains, interview questions, fieldnote items, or initial coding categories. Providing further detail, such as examples of observation domains, or an initial coding framework, would improve transparency and reproducibility.	The adapted conceptual model as well as the study research questions supported development of the topic guides and observation tools. These have now been provided in full as a supplemental file (as above) and will give the reader an understanding/overview of the domains. We hope that this will address the reviewer's comment.<br><br>The process of analysis is ongoing and the developed coding framework will be published with the findings (along with appropriate corresponding authorship acknowledgement by the wider team members who contributed to its development).
Outcome<br>The protocol includes a wide range of evaluation targets, including local operationalisation, barriers and facilitators, costs and benefits, unintended consequences, public awareness, activation frequency, caller characteristics, and treatment pathway changes. While this breadth is understandable for a formative implementation evaluation, the outcomes or evaluation domains appear somewhat overextended and are not clearly prioritised. Clarifying the main evaluation domains and	This is a formative evaluation of the implementation of a complex intervention, the implementation process being the primary focus of analysis rather than on outcomes/impact/effectiveness. As such, the main domains are the local operationalisation of Martha's Rule and the barriers and facilitators affecting this process, whilst ensuring equity is a focus. The secondary domains are existing models, public awareness, activation frequency and caller type, costs and benefits as well as unintended consequences. This detail has

distinguishing core outcomes from exploratory or contextual measures would improve clarity and interpretability.	been added to the paper under the research questions.
Statistical analysis<br>Several aspects of the quantitative analysis plan remain somewhat vague, including the final NHSE metrics to be used, the definitions of key variables, the criteria for conducting inferential analyses, and the handling of subgroup analyses and missing data. These points should be clarified to improve transparency.	We have revised and provided further detail on both the NHSE metric and survey analysis on page 14 of the main paper.

VERSION 2 - REVIEW

**Reviewer**	**1**
**Name**	**Koike, Daisuke**
**Affiliation**	**Nagoya University Hospital, Department of patient safety**
**Date**	**21-Sep-2026**
**COI**	

Thank you for the careful revision. The authors have adequately addressed my previous comments, and I have no further substantive concerns. I support acceptance of the manuscript in its current form.
