# Supplementary material for: National formative evaluation of the implementation of Martha’s Rule in English acute providers: an ethnographic study protocol
Source: BMJ Open. 2026 Nov 11;16(11):e118837. doi: 10.1136/bmjopen-2026-118837 (PMC13756334; doi:10.1136/bmjopen-2026-118837)
Supplement: Supplementary file 2 [file bmjopen-16-11-s001.docx]

### **Supplementary Material 1**

### **Overview of procedure at sites (including consenting processes)**

The following research procedures will be followed at each participating site. The formal data collection procedures at each site are summarised in figure 2.

*Preliminary informal ‘fact-finding’ interviews:* In the early phase of the overarching study, initial informal interviews will be undertaken with trust system leaders, implementation team members, and operational and/or clinical managers to learn about the planning for implementation including any potential barriers, current practices for escalating deteriorating cases for both staff and patients and their families prior to implementation, how patients are currently supported to escalate outside of their base teams, as well as the CCO team and how it operates. Staff partaking in these interviews will be asked for their verbal permission for the interview to take place and informed that written retrospective consent will be sought on the commencement of the formal data collection phase. Researchers will take field notes during and after these interviews and, will seek retrospective consent to incorporate these fieldnotes in the framework analysis. At the same time of these fact-finding interviews, we will seek to obtain implementation-relevant strategy documents and service specifications to facilitate our understanding of the operational models and teams and their function, contextual factors relevant to implementation, as well as plans for building awareness about Martha’s Rule across the sites.

*Ward and (P)CCO observations:* We will conduct general non-participant observations on participating wards and will shadow CCO team members to observe and understand staff and team functioning and processes regarding the planning and delivery of Martha’s Rule. Where applicable, we will also shadow the PCCO coverage of at least one site by the respective ODN. Participating settings will be observed at one time point at an early stage in the formal data collection phase to gain a contextual understanding of the environment and team culture. We will also have informal conversations with staff to gather information on these aspects of working and will take anonymised fieldnotes of these observations. Following general observations on wards, researchers will conduct focused intensive observations of clinical activities on wards (e.g. ward rounds, safety huddles, etc) at two separate timepoints over the life of the evaluation (i.e. a minimum of four focused observation periods at each site). We will select the clinical activities to be observed in consultation with managers during initial meetings to obtain their permission to conduct the study on their ward. At the start of the observations, the study and the purpose of the focused observations will be explained and verbal permission obtained from all stakeholders present – a record of this will be kept in fieldnotes. Similarly, opportunities to shadow (P)CCO team members will be arranged at two timepoints over the course of the evaluation to gain an understanding of the procedures followed in relation to Martha’s Rule (e.g. triaging of calls to the local escalation service, clinical response to patient, liaison with ward staff, etc.). No identifiable details will be recorded in the structured fieldnotes from these observations.

At each setting where observations are being conducted, we will display posters to explain the nature of the study, as well as the observations and interviews being conducted. This poster will contain a QR link giving access to information sheets outlining the purpose of the observations, and an opt-out clause. Verbal permission will be obtained from all present at the time of each intensive focused observations of clinical activity. We will stress confidentiality and anonymity of staff opinions and decisions taken and will not make notes of patient identities or any personal information about patients or their treatments that could identify them.

*Patient and family recruitment and participation in interviews:* Potentially eligible patient and family interview participants will be identified during observations with the help of present ward staff and/or nurse in charge (based on inclusion criteria and purposive sampling needs such as diversity) and introduced to the researcher who will inform them about the study. Ward staff and researchers will approach patients and family members during or directly following observations to invite them to participate in a brief follow up interview to be asked more about the relevant healthcare experience and about their awareness of Martha’s Rule. Once identified, a potential participant will be given a flyer about the study by the researcher and asked to complete a brief screening questionnaire. If inclusion criteria for the study are met, a detailed information sheet and a consent form will be provided to the potential participant. After allowing as much time as needed for potential participants to consider and make a decisional about the study, those willing to participate will be consented by the researcher. As data collection continues, participants will be purposively selected based on diversifying the sample using the screening data provided (see section on purposive sampling).

In addition, a smaller number of interviews will be conducted with those patients and family members escalating concerns to CCOs using the local escalation service under Martha’s Rule (e.g. Call4Concern). CCO administrators and/or the local primary investigator will use electronic lists of patients or family members contacting CCOs/local escalation service to contact them (via phone or in person) to briefly explain the purpose of the evaluation and the interviews being carried out with a researcher. Patients and family members from these lists will be given a flyer and/or business card about the study which includes the researchers’ contact details. This may be given during their inpatient stay or posted following discharge as most appropriate. Researchers will be available to support this process but will not have access to identifiable patient data. Patients and family members can contact the researchers directly or give permission for their contact details to be forwarded to the research team. Researchers will complete the screening questionnaire with potential participants, and if selected for interview, will provide participants with information sheets and obtain informed consent.

Interviews with patients and family members will take place on site where it is possible to ensure privacy and confidentiality (e.g. private ward room) or undertaken remotely following discharge.

*Staff recruitment and participation in interviews:* Following observations, a small number of interviews with staff may be conducted to fill in any gaps in understanding (from informal conversations during observations) of relevant clinical procedures, culture, roles, etc. A sub sample of staff involved in the management and delivery of care and support (identified through the preliminary interviews described above and those participating in observations) will be given information about the interviews and invited to take part in an individual face-to-face or remote interview about their work with a member of the research team. Those participating will be asked to complete a questionnaire about their demographic details. We may ask a few staff to complete a second follow-up interview towards the end of the evaluation where it is necessary to gain understanding of progress in the process of implementation. We will also interview a small number of implementation team and strategic leaders in each participating site.

All patients, family members and staff will be provided with information about the study at the time of invitation to participate. Written informed consent will be sought from all participants participating in interviews except where this is not possible and recorded verbal consent may be necessary.
