# Supplementary material for: National formative evaluation of the implementation of Martha’s Rule in English acute providers: an ethnographic study protocol
Source: BMJ Open. 2026 Nov 11;16(11):e118837. doi: 10.1136/bmjopen-2026-118837 (PMC13756334; doi:10.1136/bmjopen-2026-118837)
Supplement: Supplementary file 3 [file bmjopen-16-11-s002.docx]

# **Supplemental Material 2**

**Formative Evaluation of Martha’s Rule – Observation tools**

**Tool 1. General Ward Observations**

**Part 1 – General Observation Notes**

| **Site (anonymised name):** |  |
| --- | --- |
| **Ward:** |  |
| **Date and Day of week** |  |
| **Time started:** |  |
| **No. of hours observed:** |  |
| **Researcher:** |  |

Observation notes (e.g. physical context, number of occupied beds, staff ratios, ambiance, sounds, pace of work, clinical procedures performed, etc). Include vignettes where possible.

Reflections

**Tool 2. MR1 Focused Observations_Clinical Meetings and other relevant clinical events**

Part 1 – Observation details

| Site (anonymised name): |  |
| --- | --- |
| Ward: |  |
| Date and Day of week: |  |
| Time started: |  |
| No. of hours observed: |  |
| Researcher: |  |
| Type of meeting/clinical event observed: (circle) | Ward Round / Handover / Safety Huddle / Medication Round  Other meeting (specify) _________________  Other clinical event (specify) _________________ |

Please note, observation notes should maintain the anonymity and confidentiality of individuals present and/or being discussed. Please do not record names or identifiable details of any individual, including the discussion of any patient cases during these meetings.

Part 2 – Participants and consent

Please introduce self and briefly explain: the purpose of the observation (to observe relevant clinical meetings or clinical events in relation to Martha’s Rule), participation is voluntary, that confidentiality will be maintained, and no identifiable patient details will be recorded. Ask for verbal consent to undertake the observation. If any person present declines participation, the observation should be terminated.

| Staff present (number) :  Indicate verbal consent taken with a tick in box | Num Consented (mark x)   - Ward manager ( ) - Nurse-in-charge ( ) - Nurse ( ) - Consultant ( ) - Registrar ( ) - Junior doctor ( ) - HCA ( ) - AHP (specify) _________ ( )   (specify) _________ ( )   - CCO/RRT member ( ) - Patient ( ) - Family member ( ) - Other (specify) ______ ( ) - Other (specify) ______ ( ) |
| --- | --- |

Part 3 – General contextual details and, where relevant, meeting structure

Contextual notes (Please note details that are relevant to the type of observation: physical context, type of clinical event, pace of meeting, leadership/chairing, purpose of the meeting, clinical discussion, sequence of meeting (e.g. order of types of patients, bays, etc. discussed), administrative/management/staffing items, etc)

Part 4 – Observations in relation to MR1

Please record notes relevant to MR1, any staff identification of deterioration and the escalation of concerns.

| MR1 observation notes |  |
| --- | --- |
| A. Expression of concerns:   Record expressions of concern about patients; how these are conveyed and to whom; how these are responded to and any decision-making that follows. |  |
| B. Identifying deterioration:   Record current clinical information used to obtain deterioration info; If applicable, record how deterioration was discussed, and the plan of action, etc. |  |
| C. Escalation:   Record any escalation processes and how they were discussed; what factors were considered in escalating concerns, escalation ‘pathway’ through the staff structure, outcome of the discussion, etc |  |
| D. Martha’s Rule 1:   Record any consideration of using the local escalation helpline, decision-making, and how this was carried out (e.g. using helpline, contacting the CCO in another way, etc) or contacting another team to provide review or advice? |  |

Part 5 – Follow-up conversation with staff. Where there was no discussion of escalation during the clinical meeting/event, please have a conversation with a member of staff to understand the usual escalation routes should it be needed . Please record any relevant notes here based on the guidance A-D in Part 4.

Part 5 - Other relevant field notes

Part 6 - Reflections

**Tool 3. MR 3 Focused Observations_Structured Wellness Question**

Part 1 – Observation details

Please note, observation notes should maintain the anonymity and confidentiality of individuals present and/or being discussed. Please do not record names or identifiable details of any individual.

Special instruction for PAEDS WARDS: Minimum age of eligibility for the study is 18yo. Any child or young person below this age will not be consented into the study, and should only be approached for observation when a parent or guardian is present. The parent or guardian will be asked to give consent for participation in the study and any subsequent semi structured interview.

| Site (anonymised name): |  |
| --- | --- |
| Ward: |  |
| Date and Day of week: |  |
| Time started: |  |
| No. of hours observed: |  |
| Researcher: |  |
| Type of clinical activity observed: (circle) |  |
| Staff member observed: | Specify role: ______ |

Part 2 – Observation and consenting procedure

Pre-observation: Prior to starting observations, please liaise with the staff member asking the wellness question (e.g. PWQ) to explain the study, seek their consent (tick box below), and set up the observation. Ask staff to assist you to introduce yourself to each patient/parent guardian and seek their verbal consent for the observation.

| Indicate verbal consent from staff member with a tick in the box | Consented (mark x)  Staff consent (specify job role) ______________  Other staff member (specify role) _________________ |
| --- | --- |

Observation procedure: Please introduce self and briefly explain: the purpose of the observation (to observe how the member of staff is asking questions related to Martha’s Rule), participation is voluntary, that confidentiality will be maintained, and no identifiable patient details will be recorded. Ask for verbal consent to undertake the observation. If any person present declines participation, the observation should be terminated. Please note, parent/guardian should provide consent for participation where a child or young person under 18yo is involved. Following the observations, patients or family members can be recruited for interview. Please liaise with staff to identify suitable participants.

Part 3 – General process notes on discussion with staff member prior to observation

Clinical routine notes

Please ask staff member how they would usually conduct the clinical activity/routine under observation. Record observation notes on: the purpose of the clinical activity under observation, number of bays/beds to be observed, usual sequence of activities routinely carried out during such clinical activity/routine, how the structured wellness question is asked and recorded, use of any charts (include a photo of the tool with permission), etc )

Part 4 – Observations in relation to MR3

Please record notes relevant to MR3, pertaining to the structured method for obtaining wellness information.

Patient No._____

Bay No. _____ Bed No.______

Patient: Medical condition/reason for admission ______________ Length of stay on ward (days) _______________

| MR3 observation notes |  |
| --- | --- |
| Verbal consent:  Please record verbal consent for observation. | Num Consented (mark x)   - Patient ( ) - Parent/guardian ( ) - Other (specify) _________ ( ) |
| E. Clinical process   Record details of the clinical process carried out by staff in general and note at what point in the activity that the wellness question was asked |  |
| F. Structure wellness question:   Record how the structured wellness question was asked (verbatim if possible), when and to whom it was asked (Patient/parent); the patient’s response (without identifiable details) and how this was recorded on the EPR system, any relevant follow up questions; if applicable, record notes of any discussion that followed relating to the patient’s condition, etc. | 1. Structured question, as asked by staff: ____________________________ 2. Follow up question (if any): ______________________________________ 3. What was recorded on the Electronic Patient Record system for the wellness question: ____________________________________ |
| G. Worries and concerns   Record any expressions of concern from patients and/or family member; how these are conveyed and when (e.g. at the start, following structured wellness question); |  |
| H. Plan of action following wellness question     Record how any concerns are responded to by staff member. |  |
| I. Escalation (by staff, patient, family):   Record any escalation processes and what factors were considered; how, if at all, escalation was discussed with the patient/family member; record any consideration of using the local escalation helpline, and how this was carried out (e.g. using helpline, contacting the CCO in another way, etc) |  |

[DUPLICATE PART 4 for each additional patient being observed]

Part 5 – Follow-up conversation with staff. Please ask staff the questions in the box to facilitate this conversation. These can be rephrased and tailored to your own style.

- What do you know about Martha’s Rule? What do you think about Martha’s Rule being rolled out on your ward?
- How do you feel about asking the PWQ/wellness question to your patients? (Prompts: How helpful is it? (e.g. building and maintaining your relationships with patients, meeting patient needs, in identifying deterioration) Have there been any challenges for you? How have you overcome these?)
- Have you had to adapt the question yourself to suit your needs or personal ways of working? (Prompt: how so?) And for particular patient groups? (Which groups and how so?)
- If you needed to use the helpline to escalate your concerns, how confident would feel to do so? (Prompt: what affects your confidence? Have you had to use the service? If so, can you tell me briefly about it?)

Part 6 - Other relevant field notes

Part 7 - Reflections

**Tool 4. CCOT Shadowing General Observations**

Part 1- Observation details

| Site (anonymised name): |  |
| --- | --- |
| CCOT (name, where there are two or more, please specify) |  |
| Date and Day of week: |  |
| Time started: |  |
| No. of hours observed: |  |
| Researcher: |  |
| Staff member observed | Specify role: _________________ |

Part 2 – Observation and consenting procedure

General guidance for conducting CCO observations:

CCO observations usually involve shadowing an individual member of the team (in some teams, this will be the member holding the phone) for up to the duration of one shift. In some teams, there may be a co-ordinator who handles the calls as well as clinical staff who respond to calls – in these instances two members (admin and clinical) can be consented for the observation to allow us to generate a full picture of the processes. In sites where a site matron is holding the phone, the team will also shadow this staff member to observe how the calls are received and then referred to the CCO etc. Staff will not be shadowed during breaks or for any (confidential) meetings.

Pre-observation:

Where possible, ascertain in advance when Matha’s Rule call rates are likely to be higher. Where there is some variation in call rates, observations will be arranged for periods where calls under Martha’s Rule are more likely. It will also be necessary to identify the appropriate member(s) of the team to shadow on the day of the observation before scheduling.

CCO observations will need be set up with individual members of the CCO team in advance so that they are aware and can consent to being shadowed for up to the full duration of their shift. Prior to starting observations, and once you’ve introduced yourself, please liaise with the staff member you are about to shadow and ask some initial questions to help you will in the relevant sections below (eg. part 4).

As with all other observations, staff being observed should be informed that participation is voluntary, that confidentiality will be maintained, and no identifiable patient details will be recorded. Ask for verbal consent to undertake the observation.

Interviews:

Where possible during quiet periods in the observation, CCO staff can be invited to and complete an interview. They would need to provide written consent in the usual way as these interviews are separate from the observation itself. Interviews can be paused and recommence if there are incoming calls to attend. Otherwise, a time to complete the interview can be arranged on another occasion.

Part 3 - General team and contextual details

Please note details such as number of staff members on duty, physical environment and location of the team office, wards and units the team serves noting, where relevant, other CCOTs in the trust and site(s) it serves, ambiance, sounds, pace of work, etc. Some of the details will need to be obtained by asking staff.

Part 4 – Usual clinical routine and activities performed and handling of calls

Routine notes

Please ask staff member what their activities are for the day and how a typical day looks like for them. Record observation notes on the role performed by the staff for the day e.g. Admin, clinical, etc.

Calls (including bleeps)

In addition, please ask staff how many and what type of calls/bleeps are usually received on such a day, if there are any patterns they have observed (e.g. from particular wards, particular patients/patient groups, etc). Is there a voice message system in place? If so, in what timeframe must calls be responded to under MR and how does this usually work.

Part 5 - Team culture

Please record observation notes on the various roles and functions of the team members, their ways of working together, the team culture, dynamics, hierarchy etc.

Part 5 - Reflections

Reflections

**Tool 5. CCOT Clinical Observations**

Part 1- Observation details

| Site (anonymised name): |  |
| --- | --- |
| CCOT (name, where there are two or more, please specify) |  |
| Date and Day of week: |  |
| Time started: |  |
| No. of hours observed: |  |
| Researcher: |  |
| Staff member observed | Specify role: _________________ |

Please note, observation notes should maintain the anonymity and confidentiality of individuals present and/or being discussed. Please do not record names or identifiable details of any individual.

In advance of shadowing staff as they respond to calls, attend meetings etc, please inform the CCO member that they can ask us not to observe any events/meetings. Please also ask the CCO staff member to introduce you to the ward manager/nurse in charge (where relevant and this is possible) and to anyone present when attending to the patient (patient, family, other staff). Either you or the CCO staff can then explain that we are observing how the CCO staff respond to calls and that we will not be collecting any information that would identify any individuals. You should have to hand the information sheets for observations in case anyone would like further information. If any individual does not wish to be observed it the observation of that event should be terminated.

Part 2- Observation of calls to the CCOT/Site Matron

Call no.____

Mode of ‘call’ (Delete as necessary): Phone call to general CCO number / Martha’s Rule helpline / Site matron / bleep / app / website / Other (please specify) ________________

Time of incoming call _______. Duration of call ________

Voice message left: Yes/No, Time message picked up. _____ Response/call back time ________

| Observation notes |  |
| --- | --- |
| A. Who is the caller? | - Patient - Family/friend - Staff (specify position) _______________ - Other (specify)____________ |
| B. Are they previously known to the CCO?   If yes please give a brief summary of relevant details of CCO involvement in their care. | - Yes - No |
| C. Where is the patient located?   Please note the ward details and record whether this information was given by caller or looked up by CCOT staff using patient records. | Ward _____________  Other location ______________  How was the information obtained? |
| D. Concern(s) of the caller   Record the main expressions of concern from the caller noting when the concerns started and when they decided to make the call. Where possible, record concerns verbatim. Please note whether it was possible to hear the caller directly or whether the CCO staff conveyed the information to you following the call.  Where this is observed, please note any observations of emotion, distress or abuse towards the staff member and how this is dealt with and responded to be the CCO staff. | Main concerns of the caller:  Additional contextual notes (emotion or any instances of abusive behaviour towards staff): |
| E. Plan of action following the call   Record how the concerns were responded to by the staff member (using tick boxes),  If the CCO staff has decided to carry out an immediate review Part 3 will also need to be completed.  When possible, ask about the CCO staff member’s decision-making process in responding to the call and what factors were considered.  Record the summary of the intervention given by CCO staff. Noting any timescales for intervention given by staff. | - Phone advice - Referral to another service/ODN - Responded to directly by the CCO team member - Other escalation (please specify)____________   Notes on the decision-making process of the staff member:  Summary details of the intervention |
| F. Call type (please tick box and complete notes under questions)   Was this call categorized as a Martha’s Rule call or other? Please specify and ask staff if it is not apparent from the call. Ask staff why this was categorized in that way. | - General call/bleep to CCOT - Call/bleep under Martha’s Rule - Misc/Other (specify)____________   Please specify how, if at all, the CCO staff distinguishes the type of call:  For calls under Martha’s Rule, how does the CCO staff member decide if this is an ‘appropriate call’? |
| G. How was this call documented?   Have a conversation with the staff once they have come off the phone and are available to talk. | How, if at all, is the type of call recorded and on what system is it recorded: |

Part 3 - Observation of CCO response arising from call to helpline

Patient: _________. Condition: _____________

Ward: ______________

Time: __________

No. of hours observed: ______________

Was this a Martha’s Rule call? 🞎Yes	🞎No

Please remind the staff member that you’re shadowing to introduce you where this is possible (non-emergency calls), and briefly explain: the purpose of the observation (to observe how the CCO member responds to calls for a study about Martha’s Rule), participation is voluntary, that confidentiality will be maintained, and no identifiable patient details will be recorded. If any person present wishes for us not to observe, the observation should be terminated.

| Observation notes |  |
| --- | --- |
| Verbal consent:  Please record verbal consent for observation | Num Consented   - Patient (if possible) ( ) - Parent/guardian ( ) - Other (specify) _________ ( ) |
| A. Initial response   Please record who the CCO team approach initially (caller/ward staff/ etc) and how they manage the communication between patients/family and staff. Also note any additional clarification of main concerns as well as any information about how the issue was identified. For example, in cases of deterioration, did a change in the PWQ trigger the escalation – this will be relevant in wards participating in the pilot rollout. Please also note any views from the other party (patient) that might contrast with the caller’s. |  |
| B. Clinical assessment process and tasks   Record details of the clinical assessment process carried out by CCO staff as a result of the escalation. |  |
| C. Decision-making and outcome   Record the decision-making of the CCO staff member as well as the outcome of the call e.g. changes made to current treatment plan, referral or advice given. |  |

Part 4 - Other relevant field notes

Part 5 - Reflections
