# Supplementary material for: National formative evaluation of the implementation of Martha’s Rule in English acute providers: an ethnographic study protocol
Source: BMJ Open. 2026 Nov 11;16(11):e118837. doi: 10.1136/bmjopen-2026-118837 (PMC13756334; doi:10.1136/bmjopen-2026-118837)
Supplement: Supplementary file 4 [file bmjopen-16-11-s003.docx]

**Supplemental Material 3**

**Formative Evaluation of Martha’s Rule - Interview Schedules**

These versions of the interview schedules are indicative of the nature and content of the interviews with participants recruited from sites. Researchers will use the schedules flexibly to frame the questions and ask prompt questions based on their developing understanding of the participant’s experience and views. The schedule has been developed by the research team based on existing literature and the conceptual framework for the evaluation, and where relevant, edited with input from PPI partners. Schedules were piloted with a small number of participants at the start of data collection and refined for use in subsequent interviews.

**Interview Schedule for Patient and Family Participants**

1. **Introduction**  (5 minutes):

*(Aim: to introduce the study and interviewer)*

*Ask the participant if they have any questions before commencing the interview.*

***[Researcher: please adapt the questions for family members partaking in the interview as appropriate, for example, by substituting ‘your’ with ‘your loved one’]***

2. **Opening question**

*(Aim: To understand brief overview of the participant’s inpatient experience)*

- Please can you briefly tell me about your current/recent experience as an inpatient on [name of ward]. (Prompts: What were your feelings toward staff? To what extent did you have confidence in their ability to provide the treatment required? How safe did you feel on the ward?)

3. **Research topics**

*(Aim: Questioning phase to explore and expand on pertinent aspects of the respondent’s experience of being asked about their condition, their awareness of and views on Martha’s Rule, as well as any decision about escalating their concerns to [local escalation service])*

A. Experience of being asked about their condition (MR3)

- What did you think about being asked by staff about your well-being or condition on a regular basis? (Prompts: How helpful, if at all, was this question to you personally in thinking about your symptoms and condition? What did you think about the frequency with which you were asked this question?)
- How did being asked this question make you feel? (How, if at all, did it affect your relationship with staff? Did the staff member follow up with more questions about your condition or wellbeing? What did they do with the information you gave (e.g. record, refer to a clinician, etc)?)
- Did you experience any changes in your condition during your stay?
  - If so: What changes did you experience? How did you realise these changes? Did being asked about your condition help you to realise these changes? What, if anything, did you do when you realised your condition had changed?
  - If not: If you were getting worse and had a problem with the treatment or care that you were getting or you wanted to talk to someone about it, would you have known who to contact?

B. Awareness of Martha’s Rule and [local escalation service e.g. Call4Concern helpline] (MR2&3).

- Have you heard of Martha’s Rule?

*If participant answers yes move directly to questions in section B1. If participant has not heard about Martha’s Rule, briefly explain the three components of Martha’s Rule and name of the local escalation service (*e.g. Call4Concern)*, and move on to section B2.*

B1. How did you first learn about Martha’s Rule? What do you know about Martha’s Rule? How easy is it to understand? Can you tell me what you think about having this Rule in place? How essential is such a service to meet your needs as an inpatient? (Prompts: Its usefulness, discussed with others, its ability to meet your needs, whether there is a strong need for such as rule)

- Have you seen any information around the hospital or ward about [local escalation service e.g. Call4Concern helpline]? (Prompts: prominence on the ward, clarity about how to activate, etc)
- Did you receive any written or verbal information about this service? If relevant, when did you receive this information? What did you think about it? (Prompts: clarity of communication, usefulness, extent to which it was tailored to their individual circumstances/needs)
- What do you think about the branding/logo for Martha’s Rule? What label do you prefer, Martha’s Rule or [local escalation service]? What do you prefer about that particular label?
- If you could make any changes to improve the information you read/were given about Martha’s Rule or [local escalation service e.g. Call4Concern helpline], what suggestions would you make?

*B2. IF the participant did not know about [the local escalation service] ask the following and move directly on to section 4.*

- If you had known about [local escalation service], would you have felt able to contact or use the service? Prompts: What might have affected your confidence to do so? E.g. language, self-belief, personality, level of support from family or within the hospital. How if at all would your personal background (e.g. ethnic/religious/sexual/gender/age/impairment/ etc) affect your willingness to raise your concerns using the service (e.g. delayed or avoided)? How if at all might your personal needs impact your ability to access the service (e.g. abilitiy to use phone or app)?
- Are there any other issues in your environment outside of the ward/hospital that would affect your ability to use [local escalation service] if you needed to? (e.g. language, family and support networks, housing, financial circumstances, news coverage or social media)

C. Decision-making about using [local escalation service e.g. Call4Concern helpline] (MR2):

*[Use information collected earlier or the answer to question 1.6 from the participant’s screening questionnaire to direct the next set of questions in either section C1 or C2. If not known, find out whether the participant* chose to contact [local escalation service e.g. Call4Concern helpline*] before proceeding.*

*C1. IF* the participant knew about but chose not to use [local escalation service e.g. Call4Concern helpline]:

- Did you think about contacting [local escalation service] at all? If not, what was the reason? If so, what reason caused you to think about using it? At what point did you think about it (e.g. at the time of deterioration or another time)? What reasons, personal beliefs, or other people influenced the decision not to pursue this further?)
- If you had wished to, would you have felt confident about your ability to use [local escalation service e.g. Call4Concern helpline]? (Prompt: What affected your confidence? E.g. language, self-belief, personality, level of support from family or within the hospital)
- How if at all has your personal background [e.g. ethnic/religious/sexual/gender/age/impairment/ etc] affected your willingness to raise your concerns (e.g. delayed or avoided)? How if at all has your needs impacted your ability to access the service e.g. hearing impaired unable to use phone)? In what way has it had an impacted?
- What, if any, other action did you take to voice your concerns about your inpatient care? (e.g. made a formal complaint, contacted PALS, a solicitor or a claims management company, charity or advice centre, shared your experience on social media

*C2. IF* the participant chose to use [local escalation service e.g. Call4Concern helpline]:

- What prompted you to contact [[local activation system e.g. Call4Concern helpline? How did you make this decision? At what point did you make this decision(e.g. at the time of deterioration or another time)? What reasons, personal beliefs, or other people influenced the decision to pursue this further? (Personal or external factors motivating decision to contact the [local activation system] e.g. family members’ influence, community peers, healthcare staff suggesting appropriateness of action)
- What was your experience of using the service to raise your concerns about the care your received? (Prompts: ease, challenges, timeliness) How confident did you feel about contacting the service? Did you make contact yourself or did you need someone to do that on your behalf?
- How if at all has your [ethnic/religious/sexual/gender] identity impacted your ability/willingness to raise your concerns (e.g. delayed or avoided raising concerns)? In what way has it had an impact?) And in your decision about whether or not to contact [local activation system e.g. Call4Concern helpline]?
- What did you think of the review of your care by the independent team (CCO)? Were you satisfied with the response? Did it meet your needs? Was your treatment plan changed? What was the outcome for you? Were you moved to a different ward/ICU? What was the response by staff on your own ward?
- How did you find the process? Was it dealt with in a timely manner? Would you tell others about and recommend the service?
- If you could make any changes to improve the [local activation system e.g. Call4Concern helpline], what suggestions would you make?

4. **Summary** (10 minutes. However, this can be extended to ensure the interview is ended sensitively and to give the participant opportunity to discuss any concerns. The interviewer will have additional sources of support to hand and will follow protocol should the participant experience distress.)

*(Aim: to round up the interview and close)*

*Interviewer: Summarise conversation and what has been shared throughout the interview.*

Ask the participant what they feel have been the most important things spoken about. Check on the participant’s current emotional state, how they will take care of themselves after the interview, and if deemed okay to do so, ask whether they have any questions before ending the interview.

**Thank the participant and close**

**Interview Schedule for Implementation Team staff**

A. **Opening questions**
1. Please could you briefly state your role in relation to the implementation of Martha’s Rule?
2. Prior to asking questions around the implementation of Martha’s Rule, I would like to ask about what processes have been/are already in place in terms of identifying deterioration and escalating concerns , on the part of staff? And what, if anything is available for patients and family members?
3. Please can I start by asking, why is Martha’s Rule being implemented in your setting? Who decided to implement Martha’s Rule? How was the decision made to implement Martha’s Rule? What kind of local or national performance measures, policies, regulations, politics and/or societal views influenced the decision to implement Martha’s Rule?
B. **Research topics**

*(Aim: Questioning phase to explore and expand on pertinent aspects of the respondent’s views and experience)*

4. What are the goals or aims for the interventions under the Rule? To what extent are these goals/aims monitored for progress? If applicable, how will you use this information?
5. Can you describe the plan for implementing the three components of Martha’s Rule at [site name]? What is your role in the planning process? To what extent did you/the team consult other stakeholders implementing and accessing Martha’s Rule, such as staff and patients and families, when developing and implementing the intervention(s)/[local escalation service]? To what extent were the needs and preferences of the patients and families served by your organization considered when deciding to implement Martha’s Rule? Was Martha’s Rule altered to meet their needs and preferences?
6. What is your communication or education strategy for getting the word out about the Martha’s Rule? What materials/modes/venues do you use? What process are you using to communicate to staff and patients and families? For example, going to staff meetings, talking to people informally, running campaigns?
7. At what stage of implementation is Martha’s Rule at in your organization? Are there different timeframes for implementing the different components of Martha’s Rule? Will you be rolling out across all wards simultaneously or in a phased way? If in a phased way, how were ward selected (what criteria were used)? How do you think the rollout is going? Why do you say that?
8. Has Martha’s Rule been implemented according to the implementation plan so far? If not, how have things deviated from the plan? What if any obstacles have there been? If so, what has helped to facilitate the process?
9. What level of endorsement or support have you seen or heard from leaders? What level of involvement have they had so? What kind of support have they given you? How has this affected things so far?
10. Have you received outside training and support, for example, from NHSE or Health Innovation Networks, with implementing Martha’s Rule? How helpful have they been? In what ways? What could they have done to better support you in this early phase of the rollout? What could they do to improve the support going forward? What kind of information exchanges have you had with other acute providers?
11. What supports, such as online resources, marketing materials, or a toolkit, are available to help you implement and use Martha’s Rule? What is your perception of the quality of the supporting materials, packaging, and branding of Martha’s Rule for implementation?
12. What is the general level of receptivity in your organization to implementing Martha’s Rule? What steps have been taken to encourage staff to commit to using Martha’s Rule? What kind of supporting evidence or proof if any at all is needed about the effectiveness of Martha’s Rule to get staff on board?
13. How have patients and families responded to the implementation?
14. Are there any other issues that have arisen that we (the evaluation team) should look into and explore in our study?
C. **Summary** (10 minutes. However, this can be extended to ensure the interview is ended sensitively and to give the participant opportunity to discuss any concerns.)

*(Aim: to round up the interview and close)*

*Interviewer: Summarise conversation and what has been shared throughout the interview.*

Check whether they have any questions before ending the interview.

**Thank the participant and close**

**Interview Schedule for frontline staff**

A. **Opening questions**
1. Please could you briefly state your role with the trust in relation to the implementation and delivery of Martha’s Rule?
2. Please can I start by asking, what do you know about Martha’s Rule?
3. Is there a strong need for Martha’s Rule? (Prompt: Why or why not? Do others see a need for the Martha’s Rule?)
B. **Research topics**

*(Aim: Questioning phase to explore and expand on pertinent aspects of the respondent’s views and experience)*

4. How is Martha’s Rule being implemented in your ward? (Prompt: structured question about condition, identifying deterioration using this question, and escalating concerns to CCO using the [local escalation service]) At what stage are you at now in the process of implementing the Rule on your ward?
5. How do you feel about Martha’s Rule being implemented on your ward? (Prompt: Do you have any feelings of anticipation? Stress? Enthusiasm? Why?)
6. How well does asking patients and families about the patient’s condition on a daily basis fit with existing work processes and practices on your ward? Are there any challenges that you had to overcome to implement this aspect of Martha’s Rule? What kinds of changes or adaptations if any have you had to make to Martha’s Rule so it will work effectively in your setting? How effective does it seem to be from your perspective in detecting deterioration in patients who are asked the questions?
7. How confident are you that you will be able to escalate your concerns about a deteriorating patient through the [local activation service] should you feel the need? What gives you that level of confidence or lack of confidence? What helps and hinders you feel able to contact the service? How confident do you think your colleagues feel about using the Martha’s Rule? What helps of hinders them?
8. What kind of supporting evidence or proof if any at all is needed about the effectiveness of Martha’s Rule to get staff on the ward on board with using it?
9. What do influential stakeholders think of the Martha’s Rule? How if at all have these opinions filtered through to impact ward staff perspectives of Martha’s Rule?
10. How do the components of Martha’s Rule compare to other similar existing patient safety procedures or services in your organisation? (Prompt: Other processes for identifying deterioration and escalating concerns) Is there another intervention that you or colleagues would rather implement? (Prompt: Can you describe that intervention? Why would people prefer the alternative?)
11. How would you describe the culture of your ward? How if at all is it different to the culture of other wards or the organisation as a whole? In what ways? To what extent are new ideas embraced and used to make improvements in your organization? If you saw a problem in your own setting, what would you do? How does the ward culture affect the uptake and delivery of Martha’s Rule as you see it? Does it pose any challenges? How if at all has the culture made it easier to adopt Martha’s Rule?
12. What if any kinds of information and materials about Martha’s Rule have been made available to you? (Copies of relevant policy and communication materials? Internal and external information sharing; Has it been timely? Relevant? Sufficient?) Who do you ask if you have questions about the Martha’s Rule or its implementation? How available are these individuals?
13. How well do you think Martha’s Rule will meet/is meeting the needs of the patients and families served by your organization? In what ways will Martha’s Rule meet their needs? What barriers will the individuals served by your organization face to accessing and contributing to components of Martha’s Rule?
14. How well have staff responded to its implementation? Have their been any barriers when CCO staff have responded to calls from patients, families or staff on your ward?
15. What impact has delivering aspects of Martha’s Rule had on you and your job satisfaction? Are there other areas of benefit and consequences for you? And for your colleagues?
16. Are there any other issues that have arisen that we (the evaluation team) should look into and explore in our study? Or were there any other points you were expecting to cover with me that I haven’t asked you about?
C. **Summary** (10 minutes. However, this can be extended to ensure the interview is ended sensitively and to give the participant opportunity to discuss any concerns.)

*(Aim: to round up the interview and close)*

*Interviewer: Summarise conversation and what has been shared throughout the interview.*

Check whether they have any questions before ending the interview.

**Thank the participant and close**

**Interview Schedule for Organisational Leaders**

A. **Opening questions**
1. Please could you briefly state your role in relation to the implementation of Martha’s Rule?
2. Please can I start by asking, is there a strong need for Martha’s Rule? (Prompt: Why or why not? Do others key people in the trust see a need for the implementation of Martha’s Rule?)
B. **Research topics**

*(Aim: Questioning phase to explore and expand on pertinent aspects of the respondent’s views and experience)*

3. Why was the decision made to implement Martha’s Rule in your trust as part of the pilot rollout? How was the decision made? What kind of local or national performance measures, policies, regulations, politics and/or societal views influenced the decision to implement Martha’s Rule?
4. Have you/your trust set goals related to the implementation of Martha’s Rule? [If yes] What are the key goals and aims for the intervention(s)? What hopes or concerns do you have?
5. How are such goals communicated across the organization? To whom are they communicated? To what extent are such organisational goals monitored for progress?
6. What kind of evidence are you aware of that shows whether or not Martha’s Rule will work as intended in your particular trust?
7. At what stage of implementation is Martha’s Rule at in your organization? How do you think the rollout is going? Why do you say that?
8. To what extent might the implementation take a backseat to other high-priority initiatives going on now?
9. What is the general level of receptivity in your organization amongst all staff? And those implementing Martha’s Rule? What steps are being taken to encourage wards to implement Martha’s Rule?
10. What costs are being incurred to design, implement and administer Martha’s Rule? What costs were considered when deciding to implement Martha’s Rule? Did you expect to have sufficient resources to implement and administer Martha’s Rule? [If Yes] What resources were you counting on? Are there any other resources that you received, or would have liked to receive? [If no] What resources will not be available? How will this affect implementation?
11. How does the infrastructure of the organization (social architecture, age, maturity, size, or physical layout) facilitate implementation of Martha’s Rule? And how does the infrastructure hinder implementation? What if any kind of structural changes is your trust making to support the implementation process?
12. How well do you think Martha’s Rule is meeting the needs of the patients and families served by your organization? In what ways will Martha’s Rule meet their needs? What barriers will the individuals served by your organization face in taking up and participating in aspects of Martha’s Rule? How have staff responded to Martha’s Rule? How well does it meet their needs and ways of working?
13. Referring back to the goals you mentioned at the start, how are you assessing progress towards implementation or intervention goals? Which measures are you tracking? How will you use this information?
14. Are there any other issues that have arisen that we (the evaluation) should look into and explore in our study?
C. **Summary** (10 minutes. However, this can be extended to ensure the interview is ended sensitively and to give the participant opportunity to discuss any concerns.)

*(Aim: to round up the interview and close)*

*Interviewer: Summarise conversation and what has been shared throughout the interview.*

Check whether they have any questions before ending the interview.

**Thank the participant and close**

**Interview Schedule for CCOT staff**

A. **Opening questions**
17. Please could you briefly state your role in relation to the implementation and delivery of Martha’s Rule?
18. Please can I start by asking, how is Martha’s Rule, particularly the [local escalation service], being delivered by the CCO team? (Prompts: triaging calls, responding to calls, etc)
19. Is there a strong need for Martha’s Rule? (Prompt: Why or why not? Do others in you team see a need for the Martha’s Rule?)
B. **Research topics**

*(Aim: Questioning phase to explore and expand on pertinent aspects of the respondent’s views and experience)*

20. How do you feel about the [local escalation service] being delivered by your team? (Prompt: Do you have any feelings of anticipation? Stress? Enthusiasm? Why?)
21. How confident do you feel about delivering the [local escalation service]? How confident do you think your colleagues are? What gives you/them that level of confidence (or lack of confidence)?
22. How well does delivering the [local escalation service] and responding to calls fit with your existing work processes and practices? Are there any challenges that you have had to overcome in order to deliver this aspect of Martha’s Rule? What if any kinds of changes or adaptations have you had to make so that it can work effectively?
23. How effective does it seem to be from your perspective in terms of responding to patients and families concerns? How if at all has it affected relationships between patients, families and staff on wards? Have their been any challenges? How if at all has it been effective in resolving communication breakdowns between these parties?
24. How would you describe the working culture in the CCO team? How if at all is it different to the culture of other CCOs or the organisation as a whole? In what ways? To what extent are new ideas embraced and used to make improvements in your team? If you saw a problem in your within your team, what would you do? How does the team culture affect the delivery of Martha’s Rule as you see it? Does it pose any challenges? How if at all has the culture made it easier to adopt and deliver [the local escalation service]?
25. What if any kinds of information and materials about Martha’s Rule have been made available to you? (Copies of materials? Internal and external information sharing; e.g., staff meetings? Has it been timely? Relevant? Sufficient?) Who do you ask if you have questions about the Martha’s Rule or its implementation? How available are these individuals?
26. What do influential stakeholders think of Martha’s Rule? How, if at all, have these opinions filtered through to impact CCO team perspectives of Martha’s Rule?
27. How well do you think Martha’s Rule is meeting the needs of the patients and families served by your organization? What barriers will the individuals served by your organization face in accessing the service?
28. How responsive have CCO staff been to the implementation? What impact has delivering the escalation service had on you and your job satisfaction? Are there other areas of benefit and consequence for you? And for your colleagues?
29. Are there any other issues that have arisen that we (the evaluation team) should look into and explore in our study? Or were there any other points you were expecting to cover with me that I haven’t asked you about?
C. **Summary** (10 minutes. However, this can be extended to ensure the interview is ended sensitively and to give the participant opportunity to discuss any concerns.)

*(Aim: to round up the interview and close)*

*Interviewer: Summarise conversation and what has been shared throughout the interview.*

Check whether they have any questions before ending the interview.

**Thank the participant and close**
