# Supplementary material for: National formative evaluation of the implementation of Martha’s Rule in English acute providers: an ethnographic study protocol
Source: BMJ Open. 2026 Nov 11;16(11):e118837. doi: 10.1136/bmjopen-2026-118837 (PMC13756334; doi:10.1136/bmjopen-2026-118837)
Supplement: Supplementary file 5 [file bmjopen-16-11-s004.docx]

# **Supplemental Material 4**

# **Formative Evaluation of Martha’s Rule - Public Survey Questions**

This survey has been developed by the research team based on relevant literature and in collaboration with our Patient Advisory Group comprising lay members of the public with relevant lived experience. It subsequently underwent cognitive testing by our partners at Picker with a diverse sample of 10 participants and was further refined. This version of the survey will be completed by both YouGov panel respondents and Care Opinion sample in the community. The survey wording was scrutinised by YouGov to ensure it meets their quality standards, and there were some small amendments to wording thereafter. The survey will be hosted by YouGov using their survey platform and panellists, and by Picker Institute in Qualtrics using a Care Opinion sample. The survey items will be included verbatim in both surveys, however the Care Opinion sample will ask an additional set of questions, highlighted in red below.

**Introduction:**

The research is being carried out by a team of researchers from the National Institute of Health Research Quality, Safety and Outcomes Policy Research Unit ([www.qso.ac.uk](https://www.qso.ac.uk)).

Our findings will be used by national policy makers and local health care providers to improve a new service available to patients and family and friends in NHS hospitals in England.

This survey is about hospital care and asks about any concerns that patients and their family or close friends might experience during an overnight stay in hospital. Your answers will be collected for research to understand public views of safety in hospital. This includes understanding peoples’ awareness of a new service available to patients and family and friends in NHS hospitals in England when they have any concerns about the safety of care.

Responses to the survey are **strictly confidential**. Only the research team will be able to see your individual survey responses. Your answers are anonymous to the research team. For further information on the survey [please click here](https://picker.org/healthcare-survey-information-sheet/) [embedded link to info sheet].

The survey should take about **15 minutes** to complete.

If you would like someone to help you complete the survey it’s fine to ask a friend or relative to help, but please make sure the answers are from your point of view. 
 
Completing the survey is entirely voluntary and you may withdraw at any stage. 
 
Thank you for your help with this important survey.    

To continue with the survey, please click ’Next’ below.

[Care opinion sample only]: If you close the questionnaire before the end of the survey, we would still like to use the responses you give, even if you do not finish the survey. Are you happy for us to do so?

- Yes, you may use my responses if I only partly compete the survey
- No, you may not use my responses unless I fully complete and submit the survey

**Survey Items:** [Filter instructions are provided in grey text]

| **Item no.** | **Key Area** | **Survey question [filter instructions]** | **Response options** |
| --- | --- | --- | --- |
| Q1 | Past inpatient experience | Have you been a patient in hospital overnight? | - Yes in the last six months - Yes, between six months and three years ago - Yes, more than three years ago - No - Not sure |
| Care Opinion sample only Q2 | Past inpatient experience | [IF YES within last 6 months or in the last 3 years to Q1]  On how many separate occasions were you in hospital overnight during this time? | - On one occasion - Two to three occasions - Four or more occasions - Not sure |
| Q3 | Past experience of providing support to a patient | [IF, yes more than 3 years, NO, or not sure to Q1]  Have you supported a family member or a close friend when they were a patient in hospital overnight?  *By ‘supported’, we mean you were there for them during some of the time they were in hospital.* | - Yes in the last six months - Yes, between six months and three years ago - Yes, more than three years ago - No - Not sure |
| Care Opinion sample only  Q4 | Past experience of providing support to a patient | [IF YES within last 6 months or in the last 3 years to Q3]  On how many separate occasions did you support a family member or close friend who was a patient in hospital overnight? | - On one occasion - Two to three occasions - Four or more occasions - Not sure |
| Q5 | Past inpatient experience - safety | [IF YES within last 6 months or in the last 3 years to Q1 OR Q3]  How safe did you feel when you or your family member or close friend were most recently in hospital overnight?  By safe, we mean in relation to your health or condition | - Very safe - Somewhat safe - Neither safe nor unsafe - Not very safe - Not at all safe - Not sure |
| Q6 | Past inpatient experience - safety | [IF Q5 = very safe, somewhat safe, neither safe nor unsafe, not very safe, not at all safe]  What made you feel this way? | - **Nothing in particular made me feel this way** - **The following made me feel this way** (please specify): _______________ - Not sure |
| *Instructions:*  *The following questions ask how you feel about certain aspects of hospital care.* | | | |
| Q7 | MR3 – Frequency | Individual staff often look after more than one patient in hospital and carry out more than one care task.  How often do you think staff should ask a patient how they are feeling when they are in hospital overnight? | - Only when they ask for assistance - Once a day - Twice a day - Three to four times a day - Five times or more a day - Not sure |
| Q8 | MR3 - Accessibility | Hospital staff have ways to check if the health of a patient is getting worse (e.g. checking oxygen level, heart rate, etc.). Patients and their family and close friends can help hospital staff spot signs of a patient getting worse too by telling staff how well they feel.  If you thought you/your family member or close friend in hospital were getting worse, how comfortable would you feel telling staff this? | - Very comfortable - Somewhat comfortable - Neither comfortable nor uncomfortable - Somewhat uncomfortable - Very uncomfortable - Not sure |
| Q9 | MR3 - Accessibility | [IF Q8 answered]  What would affect how comfortable you would feel telling staff if you thought you or your family member or friend was getting worse? | - (Please type in response)_______[Open textbox]_____ - Nothing - Not sure |
| Q10 | MR2 – Experience of Concern | Have you ever had a worry or concern about patient care or treatment in hospital? | (Select all that apply)   - Yes, when I was in hospital overnight - Yes, when my family member or close friend was in hospital overnight - No - Not sure |
| Q11 | MR2 - Experience of Concern | [IF Yes to Q10]  What concerns did you want to tell staff about? | (Select all that apply):   - I did not want to speak up about my concerns - Delays in receiving care or treatment - Given incorrect or unclear information about treatment - Language or communication barriers (including hearing impairments) - Staff being too busy or unavailable to speak to - The way other patients were being treated by staff - Mistakes or errors in treatment or medication - Feeling more poorly or condition getting worse - Cleanliness or hygiene of the ward - Staff not following safety procedures - Lack of communication from staff - Feeling ignored or not treated with respect - Lack of privacy or dignity - Staff not listening to me or taking my concerns seriously - Lack of emotional support or reassurance from staff - Not knowing who to speak to about my concerns - Being in pain or discomfort without proper relief - Not being involved in decisions about my/my family member or friends’s care - Other (please specify): _______________ - Not sure |
| Q12 | MR2 - Experience of Concern | [If Yes to Q10]  Did you tell staff about any of these concerns? | No, I did not tell staff about any of these concerns   - Delays in receiving care or treatment - Given incorrect or unclear information about treatment - Language or communication barriers (including hearing impairments) - Staff being too busy or unavailable to speak to - The way other patients were being treated by staff - Mistakes or errors in treatment or medication - Feeling more poorly or condition getting worse - Cleanliness or hygiene of the ward - Staff not following safety procedures - Lack of communication from staff - Feeling ignored or not treated with respect - Lack of privacy or dignity - Staff not listening to me or taking my concerns seriously - Lack of emotional support or reassurance from staff - Not knowing who to speak to about my concerns - Being in pain or discomfort without proper relief - Not being involved in decisions about my/my family member’s care - Other (please specify): _______________ - Not sure |
| Q13 | MR2 – Experience of concern | [IF ‘I did not want to speak up’ selected to Q12]  What were the main reasons that you did not want to speak up about your concern(s)? | Please select up to TWO response options only.   - I was worried staff would be annoyed or offended - I did not think my concern was serious enough - I was not sure who to talk to - I felt anxious or intimidated - I felt too unwell to speak up - I did not want to make a fuss or worried about being seen as difficult - I did not feel supported by staff to speak up - I did not feel confident that I’d be listened to - There was a language or communication barrier (including hearing impairment) - I did not feel confident in staff knowledge or expertise to handle my concern - I was worried it might affect how me or my family member or close friend was treated or cared for - I had tried to speak up in the past, but nothing changed - Other (please specify): _______________ - Not sure |
| Q14 | MR2 - Trust in HCP to respond | Please tell us how well you think hospital staff generally respond to concerns from patients and their families or close friends? | - Very well - Quite well - Not very well - Not at all well - Not sure |
| Q15 | MR2 – Improve staff response | What do you think is MOST important for helping patients or their family or close friends in hospital raise concerns? | (Select up to TWO responses only):   - Clearer information on how to speak up - Easier ways to raise concerns - Faster initial responses from staff - Having a dedicated member of staff whose job it is to listen to patient concerns - Quicker action to resolve the concern after it is raised - Access to interpreters (spoken language and British Sign Langauge) - Assurance that medical care would not be affected by speaking up - Other (please specify): _______________ - Not sure |
| The National Health Service for England has introduced a new service called Martha’s Rule in a number of hospitals in England. Materials (such as posters and leaflets) advertising Martha’s Rule will be available to patients, family members and close friends on wards in participating hospitals. These include the following information:  **Martha’s Rule: Detecting Deterioration**  *[You know yourself or your loved one better than anyone. You may notice small changes before they show up in our routine measurements.*  *Telling us about these changes can help us detect deterioration early, so we can take action to prevent more serious health problems.  Tell us when a change worries you. If you notice changes which could suggest a health condition is getting worse, please tell any of the staff looking after you or your loved one. If, after speaking to the care team, you remain worried and feel your concerns are not being addressed, call for a rapid review. Phone: XXXXX XXXXXX* | | | |
| Q16 | MR2 NHSE Comms and comprehension | Martha’s Rule encourages patients, family and close friends, and staff to ask another healthcare team for a rapid review when they are concerned about themselves, a family member or a friend getting worse when in hospital. It also means that hospital staff must ask patients how they are feeling at least daily.  If you were a patient, family member or close friend, would the advertising information in blue above make it clear what Martha’s Rule is about? | - Very clear - Somewhat clear - Neither clear nor unclear - Somewhat unclear - Very unclear - Not sure |
| Care Opinion sample only  Q17 | MR2 NHSE Comms and comprehension | [IF Q16 = somewhat unclear or very unclear]  Please state why the information was not clear. | - (Please type in response)_______[Open textbox]_____ |
| Q18 | MR2 Awareness | Before today, had you heard about Martha’s Rule? | (Select all that apply):   - Yes, in the news or media - Yes, as a patient in hospital - Yes, as a friend or family member visiting someone in hospital - Yes, from someone I know - Yes, from somewhere else, please specify _________ - No - Not sure |
| O19 | MR2 Accessibility | Martha’s Rule gives patients, family and close friends access to a dedicated helpline service to raise any concerns to another healthcare team and receive a rapid review of care.  How would having access to a Martha’s Rule helpline affect your confidence to speak up about any concerns? | I would feel   - Much more confident - Somewhat more confident - Neither more or less confident - Somewhat less confident - Much less confident - Not sure |
| Q20 | MR2 Accessibility | Would any of the following things put you off or make it difficult for you to use the Martha’s Rule helpline service? | (Select all that apply)   - Not being sure if my concern was serious enough - Not wanting to bother the staff - Not wanting to question or undermine staff - Not wanting to discuss my concerns with staff from another team - Not being sure staff would take me seriously - Difficulty understanding how to use the service - Fear about getting worse treatment from staff - My physical condition including disability - My mental health condition including disability - Lack of access to necessary technology (e.g., phone, internet)Language or communication barriers - Other (please specify): _______________ - No, nothing would put me off or make it difficult for me to use the service - Not sure |
| Q21 | MR2 Support and safety | How likely will Martha’s Rule make you feel safer if you are a patient, or visiting a family member or friend in a hospital ward in the future? | - Very likely - Somewhat likely - Neither likely nor unlikely - Somewhat unlikely - Very unlikely - Not sure |
| Care Opinion sample only  Q22 | Actual Experience of MR2 | Have you had any prior experience of using a Martha’s Rule helpline service to contact another healthcare team about your or your friend/ family member’s care? | - Yes - No - Not sure |
| Q23 | Actual Experience of MR2_Satisfaction | If you have had experience of using a Martha’s Rule helpline service to contact another healthcare team about your or your friend/ family member’s care in hospital, please tell us how satisfied you were with the response from staff. | - Very satisfied - Somewhat satisfied - Neither satisfied nor unsatisfied - Somewhat dissatisfied - Very dissatisfied - Not sure - I have not used Martha’s Rule helpline |
